# Impact of dispersion correction in DFT-enhanced anisotropic NMR for stereochemical elucidation of flexible marine natural products

**DOI:** 10.1007/s42995-025-00294-w

**Published:** 2025-05-21

**Authors:** Lu-Ping Chi, Xiao-Lu Li, Anton F. Ketzel, Armando Navarro-Vázquez, Caspar J. Schattenberg, Xiao-Ming Li, Xin Li, Han Sun, Bin-Gui Wang

**Affiliations:** 1 CAS and Shandong Province Key Laboratory of Experimental Marine Biology, Institute of Oceanology, Chinese Academy of Sciences, and Laboratory for Marine Biology and Biotechnology, Qingdao Marine Science and Technology Center, Qingdao, 266071 China; 2 Structural Chemistry and Computational Biophysics, Leibniz-Forschungsinstitut Für Molekulare Pharmakologie (FMP), 13125 Berlin, Germany; 3 Institute of Medical Sciences, The Second Hospital of Shandong University, Jinan, 250033 China; 4 Departamento de Química Fundamental, Centro de Ciências Exatas E da Natureza, Universidade Federal de Pernambuco, Cidade Universitária, Recife, PE CEP 50.740-540 Brazil; 5 Institute of Chemistry, Technische Universität Berlin, 10623 Berlin, Germany; 6 University of Chinese Academy of Sciences, Beijing, 100049 China

**Keywords:** Stereochemical elucidation, Residual dipolar couplings (RDCs), Residual chemical shifts anisotropy (RCSAs), TD-DFT calculations, Computer-assisted structural elucidation 3D (CASE-3D) approach

## Abstract

Although anisotropic NMR spectroscopy has emerged as a powerful method for determining the relative configuration of complex natural products, major challenges persist with structurally flexible molecules. In this study, we conducted a systematic comparative analysis of stereochemical elucidation, combining anisotropic NMR spectroscopy and density functional theory (DFT) calculations on spiroepicoccin B (**1**) and epicoccin V (**2**), which were characterized as thiodiketopiperazine marine natural products isolated from the deep-sea-derived fungus *Epicoccum nigrum* SD-388. For the flexible compound **2**, we compared various conformational sampling approaches, including an assessment of the quality of relative energies within the obtained ensembles. We demonstrated the critical role of dispersion correction within DFT computations to precisely account for weak non-bonded intramolecular interactions. By integrating anisotropic NMR analysis, chemical shifts, electronic circular dichroism, and DFT computations, we determined the absolute configurations and conformational ensembles for **1** and **2**, respectively, highlighting the significance of the intramolecular methyl–π interaction in stabilizing one of the conformers. Our study introduces new strategies to address conformational flexibility in the stereochemical elucidation of challenging organic molecules.

**Supplementary Information:**

The online version contains supplementary material available at 10.1007/s42995-025-00294-w.

## Introduction

Stereochemical elucidation stands as one of the central tasks in identifying a new natural product, yet it also represents a rate-limiting step. For molecules that are non-crystallizable, NMR spectroscopy has been established as the most frequently applied method for deriving the constitution and determining the relative configuration (Becke 1993; Bifulco et al. 2007; Li et al. 2023a; Morris 1986). Traditionally, isotropic NMR parameters such as NOEs provide information on proton-proton distances, while scalar (*J*) couplings depend on the dihedral angles of the respective bond and are thus useful for assigning the relative configuration of two neighboring stereocenters. More recently, comparing the experimental and DFT-computed chemical shifts has become a popular approach for determining the relative configuration (Grimblat et al. 2015; Guan et al. 2021; Marcarino et al. 2022; Willoughby et al. 2014).

Over the past two decades, anisotropic NMR spectroscopy has been established as a powerful method for determining the relative configuration and the precise conformation of challenging molecules (Aroulanda and Lesot 2022; Liu et al. 2019; Rettig et al. 2024). In particular, for molecules with stereocenters that are distant from each other or those with a high number of non-protonated chiral centers, long-range anisotropic NMR parameters such as residual dipolar couplings (RDCs), residual chemical shifts anisotropy (RCSAs) (Li et al. 2020; Nath et al. 2020) and even residual quadrupolar couplings (RQCs) (Lesot et al. 2020) have proven essential for unambiguously assigning their relative configuration. For structurally rigid molecules, the orientation of the molecules in the anisotropic phase can be described effectively by an alignment tensor, which can in principle be determined by five or more independent anisotropic NMR data.

However, major challenges persist concerning structurally flexible molecules. Here, different approaches to use the anisotropic NMR data to determine the structure and relative configuration have been proposed. One approach is to directly calculate the structures and the configuration based on the given NMR data, using floating chirality approaches such as Progressive Stereo Locking (Cornilescu et al. 2017) or restrained distance geometry procedures as implemented in the ConArch + package (Immel et al. 2018, 2022a, b; Reggelin and Immel 2021). This type of approach is computationally less demanding, however, usually only averaged structures of a flexible system are calculated rather than a full conformational ensemble. Recently, advancements in ConArch + have enabled the calculation of an ensemble of structures based on the provided structural input. Additionally, time-averaged force-field based approaches, such as molecular dynamics (MD) simulations with orientational restraints, have been proposed to account for conformational jumping and vibrational effects (Di Pietro et al. 2019; Sternberg et al. 2020; Sager et al. 2020; Tzvetkova et al. 2019). The force-field based determined structures often rely on the accuracy of the chosen force field. Another approach, more broadly applied in recent years, involves a two-step procedure. First, ensembles of structures for each possible configuration have to be pre-generated, and in a second step, these are fitted to the experimental data. These structures can be generated by standard Monte Carlo approaches or MD simulations (Sun et al. 2011). For small to medium-sized molecules, structures can be optimized at the DFT level, providing a more accurate description of the molecular structures (Liu et al. 2017, 2019; Li et al. 2023a, b). Although this methodology requires decent computational power, it generally offers better molecular geometries and relative energies of the conformational ensemble. More importantly, the DFT-computed structures can be directly used for time-dependent DFT (TD-DFT) calculations of the chiroptical properties of the chiral molecules. The comparison between the computed and experimental spectra allows for the determination of the absolute configuration.

Although a number of DFT methods have been developed, each showing specific advantages for certain applications, organic chemists and spectroscopists quite often calculate the organic structures and their energies by default using the B3LYP functional with standard double-zeta basis sets (Kruse et al. 2012). When choosing a structural ensemble based on a pre-defined energy cutoff, the number and particular structures present in the ensemble depends on the chosen DFT functional and method of structure generation. Particular protocols have been proposed, for fitting of DFT computed chemical shifts, such as those published by Willoughby et al. (2014) or Hehre et al. (2019). Important here is the need for extended energy cutoffs in situations with large uncertainties in the relative energies of the different conformations in the considered ensemble, such as those arising in the presence of a competition between internal and external hydrogen bonding (Navarro-Vázquez 2020). The examples in the present study highlight the necessity of a careful evaluation of the energy cutoff and the necessity of empirical dispersion methods for proper evaluation of conformational energy differences in the presence of weak intramolecular interactions.

In the present study, we determined the absolute configurations of two new identified unusual thio-diketopiperazine (TDKP) derivatives: spiroepicoccin B (**1**) and epicoccin V (**2**), isolated from the deep-see-derived fungus *Epicoccum nigrum* SD-388. TDKPs have garnered attention from medicinal chemists and in drug discovery due to their potential pharmacological properties, including anticancer, antiviral, antibacterial, and antifungal activities (Chi et al. 2020; Mayer et al. 2021; Niu et al. 2017; Song et al. 2021). Using anisotropic NMR spectroscopy, we unambiguously assigned the relative configurations of **1** and **2**, while the comparison between experimental and computed ECD spectra enabled the determination of their absolute configurations. Particularly for flexible compound 2, we generated several ensembles of structures using different DFT methods, some of which considered dispersion correction. While in most previous studies on the stereochemical elucidation of natural products based on the anisotropic NMR data, DFT has been used mostly without dispersion correction. It has been shown in many theoretical studies (Antony and Grimme 2006; Řezáč 2022) that dispersion correction, e.g. D3(BJ) (Grimme et al. 2016), can drastically enhance the performance of DFT in modeling non-covalent interactions. Moreover, compared to conventional molecular mechanics and DFT optimizations, the CREST/CENSO (Bursch et al. 2022; Grimme et al. 2021; Grimme 2019) approach employs a semiempirical quantum mechanics method to calculate the energies after optimization in the sampling process. This approach has been shown to be much more accurate and reduces uncertainties compared to energy estimations from the MMFF94 force field, as demonstrated in previous analyses of peptides and small molecules (Ehlert et al. 2022; Řezáč et al. 2018).

We demonstrated here that only the DFT calculations considering dispersion corrections (Grimme et al. 2016) provided reasonable structural ensembles that exhibited a good fit to the anisotropic NMR data. Furthermore, we would like to emphasize the strength of CASE-3D, which, unlike other computational methods, relies exclusively on a variety of independent experimental data such as anisotropic and isotropic NMR data in selecting the conformational ensemble. Using epicoccin V as an example, we showed that these two approaches are advantageous over the conventional approach based on a combination of molecular mechanics and DFT calculations without dispersion correction.

## Material and methods

### Fungal material

The fungus *Epicoccum nigrum* SD-388, isolated from the deep-sea sediment samples collected in the West Pacific March 2015 (depth − 4500 m), was identified using a molecular biological protocol by DNA amplification and sequencing of the internal transcript spacer (ITS) region. Details on the isolation, identification, and preservation of the fungus have been described in our previous report (Chi et al. 2020).

### Extraction and isolation

The fungal strain *E. nigrum* SD-388 was grown on a solid rice medium and afterwards exhaustively extracted with EtOAc to obtain a crude extract (75.5 g). The extract was fractionated by silica gel VLC (vacuum liquid chromatography) using solvents of increasing polarity (PE/EtOAc, 20:1 to 1:1, and then CH_2_Cl_2_/MeOH, 20:1 to 1:1) to yield nine fractions (Frs. 1–9). Fr. 4 (2.9 g) was further separated by CC (Column Chromatography) over Lobar LiChroprep RP-18 with a MeOH-H_2_O gradient (from 1:9 to 10:0) to yield ten subfractions (Frs. 4.1–4.10). Fr. 4.3 was subjected to CC on silica gel and Sephadex LH-20 (MeOH) to obtain compound **1** (2.0 mg). Fr. 5 (6.6 g) was further purified by CC on Lobar LiChroprep RP-18 and preparative thin layer chromatography to give compound **2** (2.9 mg).

***Spiroepicoccin B**** (****1****):* Colorless oil; [α]_D_^25^ − 66.7 (*c* 0.3, MeOH); UV (MeOH) *λ*_max_ (log *ε*) 201 (4.63) nm, 276 (3.63) nm; ECD (2.3 mM, MeOH) *λ*_max_ (Δ*ε*) 202 (+ 2.21), 224 (− 1.08), 249 (+ 0.20), 281 − 0.59) nm; ^1^H and ^13^C NMR data, DMSO-*d*_6_, 500 and 125 MHz, respectively, Table 1; HRESIMS *m/z* 371.1051 [M + H]^+^ (calcd for C_19_H_19_O_4_N_2_S, 371.1060).

**Table 1:** ^13^C and ^1^H NMR data for compounds spiroepicoccin B (**1**) and epicoccin V (**2**) in MeOH-*d*_4_

Position	Spiroepicoccin B (**1**)		Epicoccin V (**2**)	
	*δ*_C_ (DEPT)	*δ*_H_ (*J* in Hz)	*δ*_C_ (DEPT)	*δ*_H_ (*J* in Hz)
1	166.5 (C)	–	168.8 (C)	–
2	92.6 (C)	–	56.9 (CH)	3.39, 1H, t (4.0, 4.0)
3	41.2 (CH_2_)	3.35, d (16.7)	38.9 (CH_2_)	3.20, 1H, dd (14.0, 4.0)
		2.70, d (16.7)	–	2.82, 1H, dd (14.0, 4.0)
4	126.0 (C)	–	136.8 (C)	–
5	125.4 (CH)	7.07, 1H, d (7.5)	131.5 (CH)	7.16, 1H, d (7.1)
6	122.5 (CH)	6.85, 1H, t (7.5)	129.4 (CH)	7.23, 1H, t (7.1)
7	129.3 (CH)	7.09, 1H, t (7.5)	128.2 (CH)	7.20, 1H, t (7.1)
8	110.1 (CH)	6.72, 1H, t (7.5)	129.4 (CH)	7.23, 1H, t (7.1)
9	158.7 (C)	–	131.5 (C)	7.16, 1H, d (7.1)
1′	168.3 (C)	–	168.5(C)	–
2′	70.6 (C)	–	70.7 (C)	–
3′	39.9 (CH_2_)	3.38, 2H, d (1.5)	40.1 (CH_2_)	3.23, 2H, s
4′	122.3 (C)	–	122.2 (C)	–
5′	157.2 (C)	–	157.3 (C)	–
6′	116.4 (CH)	6.83, 1H, t (7.5)	116.4 (CH)	6.79, 1H, t (7.3)
7′	130.0 (CH)	7.13, 1H, t (7.5)	130.1 (CH)	7.11, 1H, t (7.3)
8′	120.7 (CH)	6.78, 1H, t (7.5)	120.6 (CH)	6.76, 1H, t (7.3)
9′	133.1 (CH)	7.10, 1H, d (7.5)	133.0 (CH)	7.08, 1H, d (7.3)
2′*S-*Me	13.3 (CH_3_)	2.31, 3H, s	12.1 (CH_3_)	1.47, 3H, s

***Epicoccin V**** (****2****):* White powder; [α]_D_^25^ –37.5 (*c* 0.08, MeOH); UV (MeOH) *λ*_max_ (log *ε*) 201 (4.66) nm, 283 (4.31) nm; ECD (4.2 mM, MeOH) *λ*_max_ (Δ*ε*) 203 (+ 2.98), 226 (− 1.32), 278 (− 0.49) nm; ^1^H and ^13^C NMR data, DMSO-*d*_6_, 500 and 125 MHz, respectively, Table 1; HRESIMS *m/z* 357.1264 [M + H]^+^ (calcd for C_19_H_21_O_3_N_2_S, 357.1267); *m/z* 379.1086 [M + Na]^+^ (calcd for C_19_H_20_O_3_N_2_SNa, 379.1087).

#### NMR experiments and assignment

Standard NMR experiments 1D ^1^H, ^13^C, 2D ^1^H-^13^C HSQC, HMBC, COSY and NOESY were first conducted in DMSO-*d*_6_ to compare with the published references and elucidate the chemical constitution. As the alignment medium AAKLVFF is compatible with MeOH-*d*_4_, all measurements were repeated using MeOH-*d*_4_ as solvent on a Bruker AV-III 600 MHz spectrometer at 300 K with a 5 mm TCI Cryoprobe using standard Bruker pulse sequences.

Details of measurement of experimental standard and anisotropic NMR data for spiroepicoccin B (**1**) and epicoccin V (**2**) were described in the ESI.

#### Computational section

Computational methods and determination of relative configurations for compounds 1 and 2, DFT calculation of intramolecular methyl–π interaction for compound 2, as well as determination of absolute configurations for compounds 1 and 2 using TDDFT-ECD calculation, were described in detail in the ESI.

## Results

### Assignment of compounds spiroepicoccin B (1) and epicoccin V (2)

**Fig. 1:**
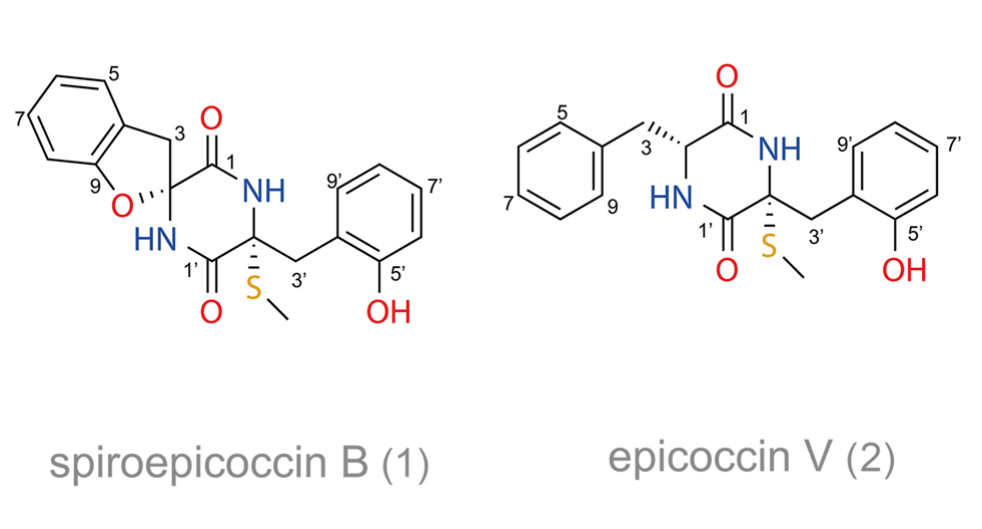
Proposed chemical structures for spiroepicoccin B (**1**) and epicoccin V (**2**)

Spiroepicoccin B (**1**), originally isolated and purified as a white powder, exhibited a pseudomolecular ion [M + H]^+^ peak at *m/z* 371.1051 by HR-ESI–MS, consistent with a molecular formula of C_19_H_18_N_2_O_4_S, suggesting 12 degrees of unsaturation. The ^1^H NMR and ^13^C NMR data (Table 1) implied a structural similarity with a previously identified spirocyclic skeleton compound, spiroepicoccin A (Li et al. 2020). A detailed analysis of 2D NMR data further confirmed the chemical constitution of spiroepicoccin B (**1**) as being shown in Fig. 1.

Epicoccin V (**2**) was found to have the elemental composition C_19_H_20_N_2_O_3_S, determined through the analysis of HR-ESI–MS (*m/z* 357.1264 [M + H]^+^, *m/z* 379.1086 [M + Na]^+^), indicating 11 degrees of unsaturation. The combination of 2D NMR data showed that compound **2** contains no spirocyclic skeleton system when compared to spiroepicoccins A (Li et al. 2020) and B (**1**), as shown in Fig. 1. The full assignment is provided in Table 1, and the procedure is described in the **ESI**.

### Measurement of experimental anisotropic NMR data for spiroepicoccin B (1) and epicoccin V (2)

We measured the one bond ^1^H-^13^C RDC (^1^*D*_CH_) and ^13^C RCSA data of compounds **1** and **2** in MeOH-*d*_4_ using the oligopeptide AAKLVFF as the alignment media (Lei et al. 2017; Li et al. 2020). The RCSA have been measured in the form of ∆∆RCSA as described in our previous publication for spiroepicoccin A (Li et al. 2020). Here, 1D ^13^C and 2D F2-coupled HSQC spectra were acquired in the isotropic state, the initial anisotropic state and the equilibrated anisotropic state, respectively. We extracted 11 RDCs and 18 ^13^C ∆∆RCSAs of compound **1**, and 15 RDCs and 19 ^13^C ∆∆RCSAs of compound **2** with sufficient accuracy. For compound **1**, three different carbon atoms were selected as references for the extraction of the ^13^C ∆∆RCSAs: (i) the carbon atom with the smallest theoretically calculated chemical shift anisotropy (CSA) (C-2′); (ii) the only methyl carbon of compound **1** which showed the smallest change in its chemical shift between the initial and the equilibrated alignment states (*S*-C); and (iii) the methyl carbon atom of the solvent MeOH-*d*_4_. Since two carbon atoms (C-2 and C-3) showed comparable small CSA values in the theoretical calculations of compound **2**, four references were applied when extracting ^13^C ∆∆RCSAs for compound **2**. The experimental details are described in **ESI**.

#### Determination of the relative configuration for spiroepicoccin B (1) using a combination of MM-based sampling, DFT free energy and anisotropic NMR data

Spiroepicoccin B (**1**) features a similar skeleton to the previously studied compound spiroepicoccin A (Li et al. 2020). They both possess two unknown stereogenic centers at C-2 and C-2′ (Fig. 1), resulting in two possible relative configurations: 2R*, 2′R* and 2R*, 2′S*. Consequently, the same two-step approach as previously proposed for spiroepicoccin A was adopted here to determine the relative configuration of **1**. We first conducted a molecular mechanics (MM)-based conformational search with a 10 kJ/mol energy window using the MMFF94 force field as implemented in the MacroModel package (Schrödinger 2020). As a result, 10 and 4 possible conformers were generated for the configurations 2R*, 2′R* and 2R*, 2′S*, respectively. These structures were further optimized at the B3LYP/6−31+G(d,p) DFT level (Becke 1993; Clark et al. 1983; Francl et al. 1982), incorporating the implicit IEFPCM solvation model (Cancès et al. 1997) for methanol. Based on the DFT-computed energies, conformers with a Boltzmann population higher than 5% were selected and incorporated in the conformational ensembles of 2R*, 2′R* and 2R*, 2′S*. Depending on whether the sum of electronic and zero-point energies or the sum of electronic and thermal free energies was used for the selection, two different structural ensembles (*RR*1 and *RR*2) were obtained for configuration 2R*, 2′R*, while for 2R*, 2′S*, the obtained ensemble remained the same. When only the zero-point vibrational energy contribution was taken into account, the *RR*1 ensemble consisted of four conformations selected for configuration 2R*, 2′R*, which differ by the pseudorotation of the benzofurane ring and the two-fold rotation of the phenyl ring around the C3′–C4′ bond. However, the computations indicated a larger preference when the phenyl ring was oriented in such a way that a hydrogen bond can form between the carbonyl group at C1′ and the hydroxyphenyl group. When free energies were used to define the population cutoff (*RR*2 ensemble), conformations with the hydroxyphenyl group placed at the opposite side of the C1′ carbonyl group were dropped from the ensemble which consisted then of two hydrogen bonded forms.

**Fig. 2:**
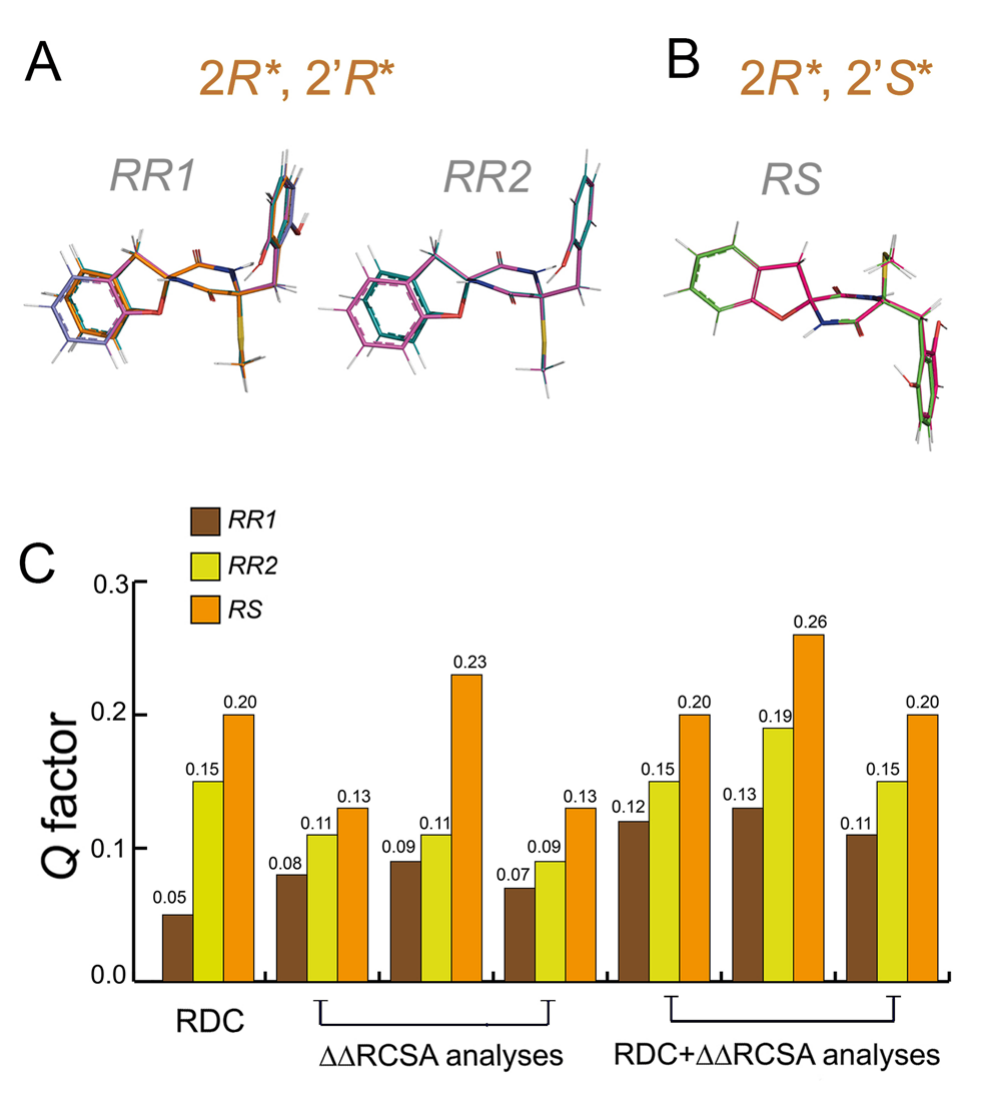
**A** Structural models generated for the 2R*, 2′R* configuration (*RR*1 and *RR*2 conformational ensembles) of compound **1**. *RR*1 indicates a structural ensemble of 4 conformers selected by the sum of electronic and zero-point energies while *RR*2 refers to the structural ensemble of 2 conformers selected by the sum of electronic and thermal free energies. **B** Structural model generated for configuration 2R*, 2′S* of compound **1**. Regardless of whether zero-point or thermal free energies were used in the selection, the same conformational ensemble consisting of two conformers was chosen. **C** Comparison of *Q* factors derived from the ^1^*D*_CH_ RDC and ^13^C ∆∆RCSA analyses for 2R*, 2′R* (*RR*1 and *RR*2) and 2R*, 2′S* (*RS*) of compound **1**. For the ∆∆RCSA analyses, the reference carbons from left to right are C-2′, *S*-C and the carbon atom in MeOH-*d*_4_

The RDC data were first fitted against the ensembles of 2R*, 2′R* and 2R*, 2′S*, assuming a single alignment tensor is sufficient to describe the orientation of both conformers (Thiele et al. 2009). As shown in Fig. 2, configuration 2R*, 2′R* exhibits a *Q* factor of 0.05 for ensemble *RR*1 and 0.15 for *RR*2. Conversely, the configuration 2R*, 2′S* yields a higher *Q* factor of 0.20. We further used the ∆∆RCSA data to distinguish between the two possible relative configurations. Similar to the analysis of the RDCs, 2R*, 2′R* showed the lowest *Q* factor, regardless of which carbon atom was chosen as the reference. Again, ensemble *RR*1 manifested a better fit to the ∆∆RCSA data than *RR*2. Furthermore, we performed a simultaneous fit of the RDCs and ∆∆RCSAs. Here, the *Q* factors of 2R*, 2′S* was about twice as large when compared to the *RR*1 ensemble of 2R*, 2′R*. Based on the clear distinction between the two possible relative configurations using anisotropic NMR data, the relative configuration of spiroepicoccin B (**1**) was determined to be 2R*, 2′R*, which is consistent to spiroepicoccin A.

#### Determination of the absolute configuration for spiroepicoccin B (1) based on ECD data

The absolute configuration of compound **1** was unambiguously determined by comparing the experimental electronic circular dichroism (ECD) spectrum and predicted spectra from time-dependent density functional theory (TD-DFT) (Autschbach et al. 2011; Pescitelli and Bruhn 2016). The comparison of the sign of the Cotton effect at the absorption bands in both the experimental and theoretical ECD spectrum reveals unambiguously the absolute configuration of compound **1** to be 2*R*, 2′*R* (**Fig. S12**), which is consistent with its analogue, spiroepicoccin A.

#### Determination of the relative configuration of epicoccin V (2) using a combination of MM-based sampling, DFT free energy and anisotropic NMR data

Similar to spiroepicoccin B (**1**), two diastereoisomeric configurations 2R*, 2′R* and 2R*, 2′S* exist for epicoccin V (**2**) due to the chiral atoms C-2 and C-2′. Initially, we employed the same approach as for compound **1** to determine the relative configuration of compound **2**.

Given that compound **2** exhibits a higher conformational flexibility than compound **1** due to the presence of a benzyl ring at C-2, exhaustive sampling of the conformational space is much more crucial in this case, as potentially many minima exist which all could contribute to the experimentally measured RDC and RCSA. If some of these conformations are neglected, agreement with the experiments will be less satisfactory, which will lead to a decreasing reliability of the determined relative configuration.

**Fig. 3:**
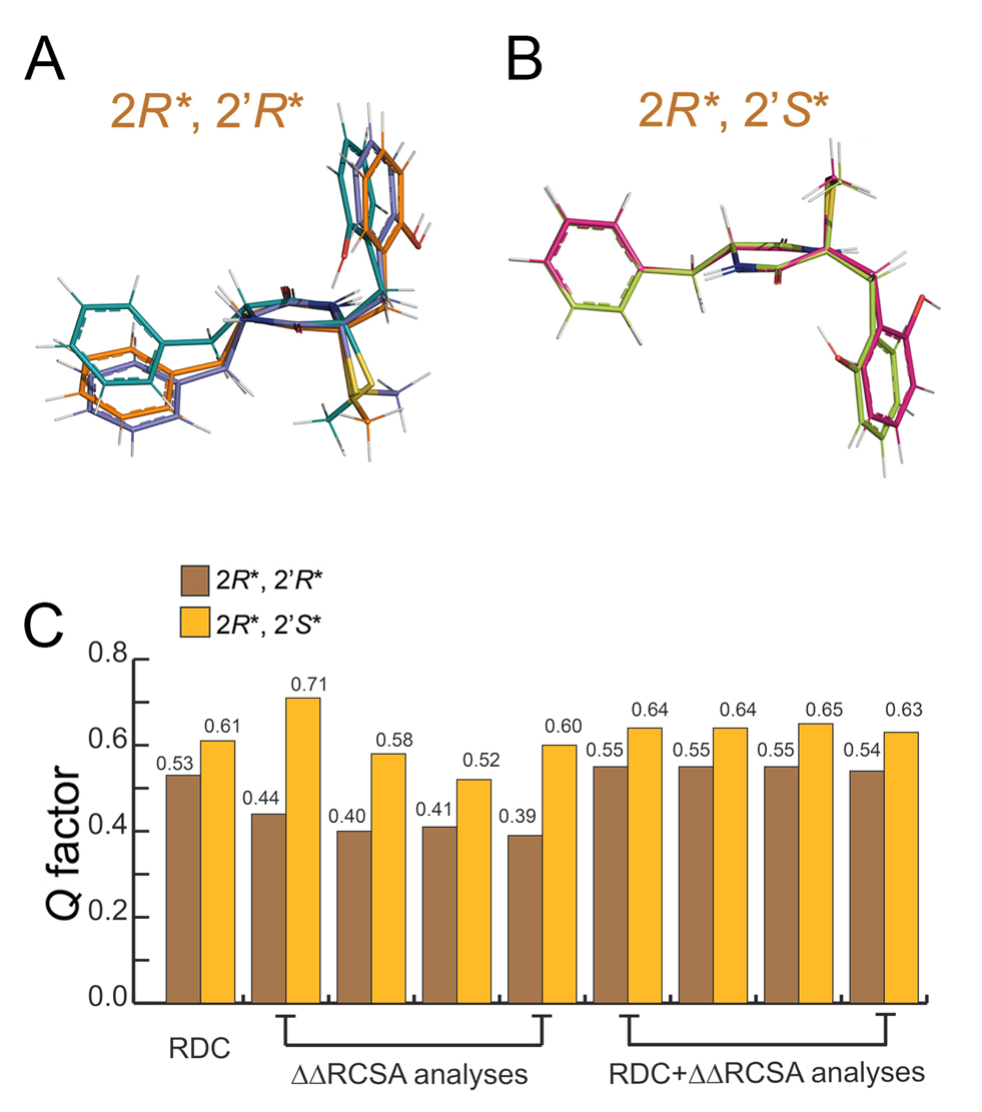
Structural ensembles of **A** configuration 2R*, 2′R* (4 conformers) and **B** 2R*, 2′S* (2 conformers) of compound **2**, selected based on the sum of electronic and thermal free energies. All conformers were optimized at the B3LYP/6−31 + G(d,p) level using the IEFPCM solvation model (methanol). **C** Comparison of the *Q* factors derived from the RDC and ∆∆RCSA analyses for the configurations 2R*, 2′R* and 2R*, 2′S* of compound **2**. For the ∆∆RCSA analyses, the reference carbons from left to right are C-2, C-3, S-C in compound **2** and the carbon of MeOH-*d*_4_

To account for the potential flexibility of **2**, a larger energy window of 30 kJ/mol was chosen as the energy cutoff in the MacroModel conformational sampling. This cutoff in particular was chosen based on theoretical benchmarks, suggesting that the error in energies using the MMFF94 forcefield can exceed 20 kJ/mol (Ehlert et al. 2022) and that increasing the energy window in CASE-3D calculations allows for a more exhaustive search of possible conformers (Navarro-Vázquez 2020). Twenty-five and 8 conformers were found for configurations 2R*, 2′R* and 2R*, 2′S*, respectively. As shown in Fig. 3, subsequent to DFT optimizations at the B3LYP/6−31+G(d,p) level and selection based on the Gibbs energy, 4 and 2 conformers which show a Boltzmann weighting of 5% or higher remained for configurations 2R*, 2′R* and 2R*, 2′S*, respectively. These two ensembles were fitted against the measured anisotropic NMR data (15 RDCs and 19 ^13^C ∆∆RCSAs), again using a single alignment tensor approximation (Thiele et al. 2009). In this case, although the configuration 2R*, 2′R* exhibited lower *Q* factors than 2R*, 2′S* when fitting the RDC and ∆∆RCSA data or a combination thereof, both configurations displayed high *Q* factors exceeding 0.3. This result indicated that the strategy of generating conformational ensembles using MacroModel and DFT optimization at the B3LYP/6−31+G(d,p) level may not be optimally suited for molecules with enhanced conformational flexibility, such as compound **2**.

**Fig. 4:**
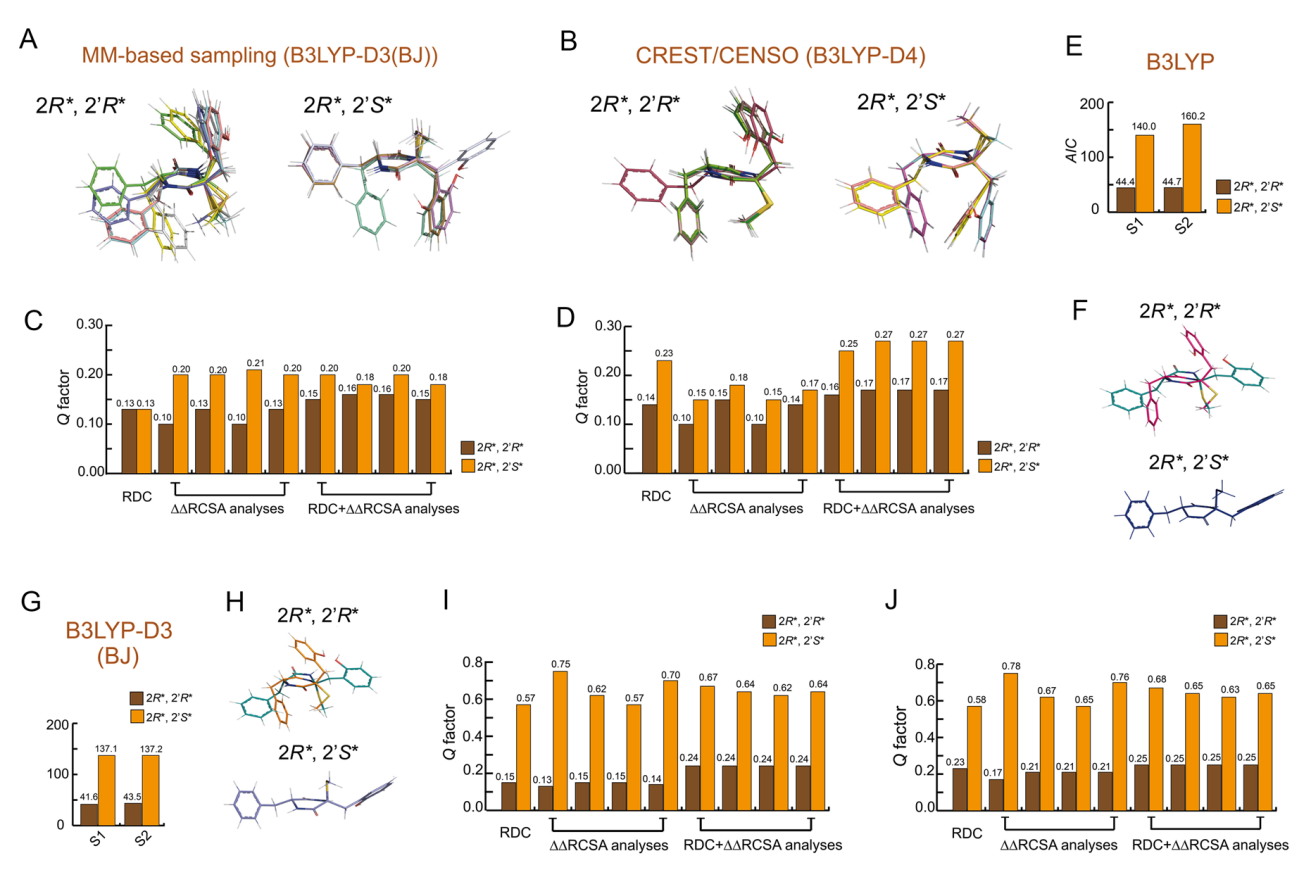
**A/C** Structural ensembles selected based on the sum of electronic and thermal free energies of compound **2** and the comparison of *Q* factors derived from RDC and ∆∆RCSA analyses. All conformers were optimized at the B3LYP-D3(BJ)/6−31 + G(d,p) level using the IEFPCM solvation model (methanol). **B/D** Structural ensembles of compound **2** selected from the CREST/CENSO method and the comparison of *Q* factors derived from RDC and ∆∆RCSA analyses. The final conformers were optimized using B3LYP-D4/def2-TZVP with the SMD methanol solvation model. **E/F/I** The *AIC* values, structural ensembles and comparison of *Q* factors derived from RDC and ∆∆RCSA analyses of compound **2** from the CASE-3D calculations without dispersion correction. (**G/H/J**) The *AIC* values, structural ensembles and comparison of *Q* factors derived from RDC and ∆∆RCSA analyses of compound **2** from the CASE-3D calculations with dispersion correction. S1 and S2 in panel **E** and **G** indicate the conformational ensembles with the lowest and second lowest *AIC* values selected by CASE-3D. In all CASE-3D calculations, the experimental ^1^H and ^13^C chemical shifts for the two possible configurations of compound **2** and the theoretical calculated chemical shifts at the B3LYP/6−311 + G(2d,p) level with IEFPCM methanol solvation with the optimized structures at the B3LYP/6−31 + G(d,p) or B3LYP-D3(BJ)/6−31 + G(d,p) level of theory were applied. In all ΔΔRCSA analyses, the reference carbon atoms from left to right are C-2, C-3, *S*-C in compound **2** and the carbon atom of MeOH-*d*_4_

In the DFT calculations described above, no empirical dispersion correction was applied, which may have led to insufficient accounting for long-range interactions within the molecules. This oversight could potentially affect the accuracy of the computed geometries and, particularly, the energies of the conformers. Consequently, we repeated the optimization and frequency calculations for all 32 conformers of compound **2**, using the B3LYP-D3(BJ)/6−31+G(d,p) level with the IEFPCM solvation model for methanol (Fig. 4A). Here, we used the D3 model for dispersion correction (Grimme et al. 2010) developed by Grimme et al*.* with Becke-Johnson damping (Becke and Johnson 2005; Johnson and Becke 2006), which helps to improve the description of short-range dispersion interactions. As shown in Table **S8**, the calculated ensembles for *RR* and *RS* based on the DFT calculation with and without dispersion correction are substantially different. The results when employing these ensembles, summarized in Fig. 4C, demonstrated notably lower *Q* factors in the RDC and ∆∆RCSA fitting compared to those obtained from DFT calculations without dispersion correction. Specifically, the configuration of 2R*, 2′R* exhibited low *Q* factors, ranging from 0.10 to 0.16 across various setups.

#### Determination of the relative configuration of epicoccin V (2) using the CREST/CENSO procedure combined with anisotropic NMR

We noted that a more sophisticated conformational sampling technique combining the Conformer-Rotamer Ensemble Sampling Tool (CREST) and the Command-line Energetic SOrting (CENSO) tool has been recently proposed by Grimme et al. (2021) and Grimme (2019). This approach integrates various DFT-based methods, offering a more automated alternative to traditional MM-based sampling and DFT optimizations. Using CREST/CENSO, we generated two Boltzmann-weighted conformational ensembles (Fig. 4B) using the DFT level B3LYP-D4/def2-TZVP with the implicit Solvation Model based on Density (SMD) (Marenich et al. 2009) for methanol. When these two conformational ensembles have been fitted to the RDC and ∆∆RCSA data, we observed *Q* factors (Fig. 4D) very similar to those obtained with an MM-based conformational search and DFT calculations that included D3 dispersion correction (as shown in Fig. 4C). Again, the configuration of 2R*, 2′R* displayed low *Q* factors, ranging from 0.10 to 0.20 across various setups, whereas the *Q* factors for configuration 2R*, 2′S* were substantially larger.

#### Determination of the relative configuration of epicoccin V (2) using a combination of CASE-3D and anisotropic NMR

Over the past decade, the computer-assisted structural elucidation 3D (CASE-3D) approach has emerged as a useful method for determining the configuration and conformation of small organic molecules based on the comparison of computed and experimental chemical shifts (Navarro-Vázquez et al. 2018). Different from DP4+ and related methods (Grimblat et al. 2015; Marcarino et al. 2022), which rely on DFT energies for conformation selection, CASE-3D fits conformational populations to available data while employing an Akaike-based model selection routine to choose the simplest conformational ensemble that best fits to the experimental chemical shifts data. This method can be further extended to incorporate various isotropic and anisotropic NMR data, including *J*-couplings, NOEs, RDCs and RCSAs (Navarro-Vázquez et al. 2018; Koos et al. 2020). To extend this study, we also employed the CASE-3D approach to determine the relative configuration of compound **2**. The 25 DFT-optimized conformers of 2R*, 2′R* and 8 conformers of 2R*, 2′S* at the B3LYP/6−31+G(d,p) level with and without D3(BJ) were used separately in the CASE-3D approach employing both ^13^C and ^1^H chemical shifts for selecting the configurations and the conformers. As shown in Fig. 4E**/**G, regardless of whether dispersion correction is applied in the DFT calculations, CASE-3D effectively discriminates between the two possible stereoisomers, with 2R*, 2′R* showing significantly lower Akaike Information Criterion (*AIC*) values. Moreover, we used the ensembles selected by CASE-3D (Fig. 4F**/**H) and fitted them against the 15 RDCs, 19 ^13^C ∆∆RCSAs, or a combination of them. The results, summarized in Fg. 4I,J, reveal a clear distinction between the two stereoisomers by incorporating anisotropic NMR data, with the configuration 2R*, 2′R* displaying low *Q* factors ranging between 0.10 and 0.25. Notably, whether the calculations were performed with or without dispersion correction did not influence the overall result, as the conformational ensembles were selected solely based on the experimental chemical shifts.

#### Intramolecular methyl–π interaction determines the main conformer in epicoccin V (2)

**Fig. 5:**
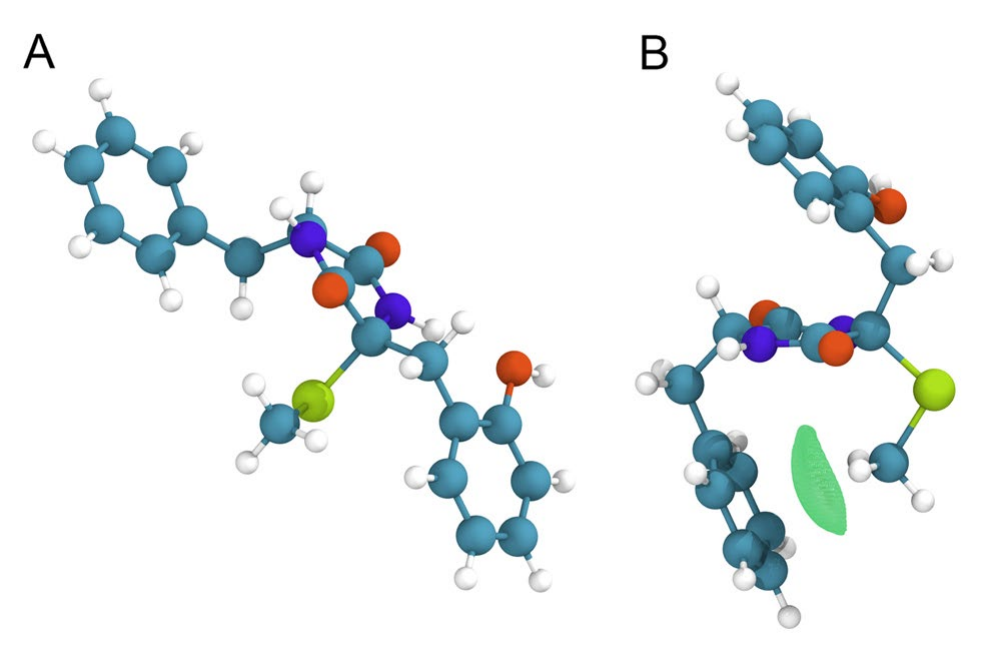
Structural ensemble of 2R*, 2′R* with the lowest *AIC* in the CASE-3D calculation showing (**A**) an extended conformer and (**B**) a more folded conformer wherein the* S*-CH_3_ group engages in an intramolecular methyl–π interaction with the aromatic ring, as indicated by the green spheres. The strength of the noncovalent interaction is visualized using the independent gradient model based on Hirshfeld partition (IGMH) (isosurface value of 0.003). More details are provided in the **ESI**

Comparing various conformational ensembles selected by different approaches, we found that two conformers are especially important for properly describing the anisotropic NMR data and chemical shifts of compound **2**. In one of these conformers (Fig. 5B), the *S*-CH_3_ group participates in an intramolecular methyl–π interaction with the aromatic ring, stabilizing this particular conformation. In the other conformer (Fig. 5A), the phenyl ring rotated away, resulting in a more extended conformation. Methyl–π interactions are frequently observed motifs in protein–ligand complexes (Umezawa and Nishio 1998; Takahashi et al. 2000; Brandl et al. 2001), and the strength of these interactions in CDCl_3_ has previously been quantified using a molecular balance approach (Carroll et al. 2011). Interestingly, in our study, this weak non-bonded interaction contributes significantly to one of the main conformers of epicoccin V (**2**).

#### Determination of the absolute configuration for epicoccin V (2) based on ECD data

Additionally, since epicoccin V (**2**) exhibits a similar experimental ECD spectra to that of compound **1**, the absolute configuration of **2** was assumed to be 2*R*, 2′*R*. For the TD-DFT calculation, the ensemble selected by CASE-3D at B3LYP-D3(BJ)/6−31 + G(2d,p) level of theory for **2** was used. The comparison between the calculated and experimental ECD spectrum confirmed the initial hypothesis that the absolute configuration of compound **2** is 2*R*, 2′*R* (**Fig. S13**).

## Discussion

We have described here the isolation, identification, and stereochemical elucidation of two rare thiodiketopiperazine marine natural products, spiroepicoccin B (**1**) and epicoccin V (**2**), produced by the deep-sea-derived fungus *E. nigrum* SD-388. Establishing relative and absolute configuration of **1** and **2** was based on anisotropic NMR data and ECD spectroscopy.

For the conformationally more rigid compound **1**, conventional force field based conformational search method followed by DFT calculation without dispersion correction was employed to generate a structural ensemble for fitting of the anisotropic NMR data. This strategy was used previously for spiroepicoccin A (Li et al. 2020), and was also successful in establishing the correct relative configuration and main conformations of compound **1**. For **1**, the presence of the two conformers differing in the orientation of the phenyl ring, seems to be essential for a good correlation between experimental and back-calculated NMR data. During the conformational sampling, we highligted the necessity of providing larger energy cutoffs when dealing with molecules presenting a competition between internal hydrogen bonding. This provides evidence for the limitations of implicit solving models to account for the competition between intra and intermolecular hydrogen bonding (Navarro-Vázquez 2020).

For the conformationally more flexible compound **2**, we evaluated various computational strategies, including MM-based conformational search, DFT calculations with or without dispersion correction, and the recently proposed CREST/CENSO method for generating conformational ensembles in an efficient and automated manner. Our findings highlighted the importance of incorporating dispersion correction in DFT calculations for accurately describing the non-bonded intramolecular interactions and energy computations. This finding was also demonstrated in previous studies (Navarro-Vázquez 2020; Řezáč et al. 2018). Nevertheless, our study relies on very limited examples and cannot be considered a benchmark study. More studies with a larger number of examples featuring different molecular scaffolds and functional groups are needed. Additionally, based on our attempt using the CREST/CENSO approach in this work, we conclude that the CREST/CENSO approach, employing the DFT level B3LYP-D4/def2-TZVP (Weigend and Ahlrichs 2005; Caldeweyher et al. 2017) which includes empirical dispersion correction, serves as an appropriate conformational sampling method for flexible molecules, such as compound **2**.

Furthermore, we compared different strategies for selecting conformational ensembles based on DFT-computed energies with an approach based on CASE-3D, wherein conformers were selected by comparing computed and experimental chemical shifts. Our results show that ensembles selected based on chemical shifts reveal a good fit to the anisotropic NMR data. Therefore, integrating CASE-3D with anisotropic NMR data could potentially reduce the impact of energy inaccuracies in DFT calculations of flexible and complex chemical systems.

In conclusion, the relative configurations of **1** and **2** were unambiguously determined to be 2R*, 2′R* using anisotropic NMR data, including RDC and ΔΔRCSA. A comparison between the experimental and TD-DFT calculated ECD spectra suggested the absolute configuration to be 2*R*, 2′*R* for both **1** and **2**. The strategies provided within this study have addressed the challenges associated with conformational flexibility in organic molecules, a persistent difficulty in stereochemical determination using NMR data.

## Supplementary Information

Below is the link to the electronic supplementary material.

The online version contains supplementary material available at https://doi. org/10.1007/xxxxxx. The Gaussian16 output files, the MSpin input and output files together with the raw NMR spectra have been deposited in Zenodo under accession code: https://doi.org/10.5281/zenodo.10986434 (DOCX 3486 KB)

## Data Availability

The data that support the findings of this study are presented within the main text and its Supplementary Information or have been deposited on Zenodo (10.5281/zenodo.10986434). Additional data are available upon request to authors.
