# Supplementary material for: Impact of dispersion correction in DFT-enhanced anisotropic NMR for stereochemical elucidation of flexible marine natural products
Source: Mar Life Sci Technol. 2025 May 21;7(4):890–900. doi: 10.1007/s42995-025-00294-w (PMC12662982; doi:10.1007/s42995-025-00294-w)
Supplement: Supplementary file 1 — The online version contains supplementary material available at https://doi. org/10.1007/xxxxxx. The Gaussian16 output files, the MSpin input and output files together with the raw NMR spectra have been deposited in Zenodo under accession code: https://doi.org/10.5281/zenodo.10986434 (DOCX 3486 KB) [file 42995_2025_294_MOESM1_ESM.docx]

Supplementary Information

**Impact of dispersion correction in DFT-enhanced anisotropic NMR for stereochemical elucidation of flexible marine natural products**

**Lu-Ping Chi ^1,^**^†^**, Xiao-Lu Li ^2,3,^**^†^**^,^*, Anton F. Ketzel ^2,5^, Armando Navarro-Vázquez ^4^, Caspar J. Schattenberg ^2^, Xiao-Ming Li ^1^, Xin Li ^1^, Han Sun ^2,5,^*, and Bin-Gui Wang ^1,6,^***

1. CAS and Shandong Province Key Laboratory of Experimental Marine Biology, Institute of Oceanology, Chinese Academy of Sciences, and Laboratory for Marine Biology and Biotechnology, Qingdao Marine Science and Technology Center, Nanhai Road 7, Qingdao 266071, China

^2.^ Structural Chemistry and Computational Biophysics, Leibniz-Forschungsinstitut für Molekulare Pharmakologie (FMP), Robert-Rössle-Str. 10, 13125, Berlin, Germany

^3.^ Institute of Medical Sciences, The Second Hospital of Shandong University, 247 Beiyuan Street, Jinan 250033, China

^4.^ Departamento de Química Fundamental, Centro de Ciências Exatas e da Natureza Universidade Federal de Pernambuco, Cidade Universitária, CEP, 50.740-540 Recife, PE, Brazil

^5.^ Institute of Chemistry, Technische Universität Berlin, Straße des 17. Juni 135, 10623, Berlin, Germany

^6.^ University of Chinese Academy of Sciences, Yuquan Road 19A, Beijing 100049, China

^†^ These authors contributed equally.

***** Correspondence:

Xiao-Lu Li

lululi666666@126.com

Han Sun

hsun@fmp-berlin.de

Bin-Gui Wang

wangbg@ms.qdio.ac.cn

**Table of Contents**

[1 Methods and Materials 3](#_Toc166516286)

[1.1 Biological material 3](#_Toc166516287)

[1.2 Standard NMR experiments and assignment 3](#_Toc166516289)

[1.3 Measurement of experimental anisotropic NMR data for spiroepicoccin B (1) and epicoccin V (2) 5](#_Toc166516290)

[1.4 Computational methods 9](#_Toc166516291)

[1.5 Determination of relative configuration for compound 1 20](#_Toc166516292)

[1.6 Determination of relative configuration for compound 2 22](#_Toc166516293)

[1.7 DFT calculation of intramolecular methyl-π interaction for compound 2 30](#_Toc166516294)

[1.8 Determination of absolute configurations for compound 1 and 2 using TDDFT-ECD calculation.^[21]^ 31](#_Toc166516295)

[1.9 Reference 32](#_Toc166516296)

[2 Additional figures of spectroscopic spectra of compounds 1 and 2 34](#_Toc166516297)

**1 Methods and Materials**

**1.1 Biological material**


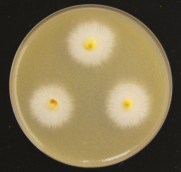


**Fig. S1** Photo of the deep-sea-derived fungus *Epicoccum nigrum* SD-388.

**1.2 Standard NMR experiments and assignment**

Standard NMR experiments 1D ^1^H, ^13^C, 2D ^1^H-^13^C HSQC, HMBC, COSY and NOESY were first conducted in DMSO-*d*_6_ to compare with the published references and elucidate the chemical constitution. As the alignment medium AAKLVFF is compatible with MeOH-*d*_4_, all measurements were repeated using MeOH-*d*_4_ as solvent on a Bruker AV-III 600 MHz spectrometer at 300 K with a 5 mm TCI Cryoprobe using standard Bruker pulse sequences.

Spiroepicoccin B (**1**), originally separated and purified as a white powder, gave a pseudo molecular ion [M + H] ^+^ peak at *m/z* 371.1051 by HR-ESI-MS, consistent with a molecular formula of C_19_H_18_N_2_O_4_S, suggesting 12 degrees of unsaturation. The ^1^H NMR and ^13^C NMR data in DMSO-*d*_6_ (**Table S1**) revealed the presence of three exchangeable protons (*δ*_H_ 8.66, 9.58, 9.58), one methyl group, two *sp^3^*-hybridized methylenes, eight *sp^2^*-hybridized methines, eight quaternary carbons (one bonded to oxygen and two amide carbonyls). The above data described all of the ^1^H and ^13^C resonances for spiroepicoccin B (**1**) and gave the structural fragments of two *sp^3^*-hybridized methylenes, one *S*-methyl group, a diketopiperazine (DKP) ring and two *ortho*-substituted phenyl systems, indicating an epithiodioxopiperazine (ETP) derivative. Detailed analysis of 2D NMR data implied structural similarity with a well described spirocyclic skeleton compound spirobrocazine C, isolated from a marine mangrove derived fungus *Penicillium brocae* MA-231 in 2016.(1) The *sp^2^*-hybridized methine group (*δ*_H_/*δ*_C_ 6.95/114.2) at C-3’ in Spirobrocazine C was replaced in **1** by a *sp^3^*-hybridized methylene group (*δ*_H_/*δ*_C_ 3.40, 3.19/37.4). Also, the upﬁeld chemical shift of the quaternary carbon C-2’ (*δ*_C_ 125.1 in Spirobrocazine C and *δ*_C_ 67.0 in **1**) and an additional *S*-methyl group indicated that the double bond was missing and the *S*-methyl group was attached to C-2’, which was confirmed by the HMBC correlations from *S*-methyl to C-2’ and from H-3’ to C-1’, C-2’, C-5’ and C-9’ as shown in **Fig. S2**. Thus, the chemical constitution of compound **1** was established as depicted in **Fig. 1** in the main text.

The elemental composition of compound **2** was determined as C_19_H_20_N_2_O_3_S through the analysis of HR-ESI-MS (*m/z* 357.1264 [M + H] ^+^, *m/z* 379.1086 [M + Na] ^+^) and NMR data in the solvent DMSO-*d*_6_ (**Table S1**), indicating 11 degrees of unsaturation. Detailed analysis of its ^1^H and ^13^C NMR data accounted for three exchangeable protons (*δ*_H_ 8.12, 8.35, 9.49), one methyl group, two *sp^3^*-hybridized methylenes, one *sp^3^*-hybridized and nine *sp^2^*-hybridized methines, and six quaternary carbons (one bonded to oxygen and two amide carbonyls). In conjunction with 2D NMR spectra, compound **2** was found to be similar with eutypellazine J, a previously reported DKP alkaloid from the deep sea derived fungus *Eutypella* sp. MCCC 3A00281 in 2017, (2) except for a missing *S*-methyl group. Instead, additional signals of a methine group (*δ*_H_/*δ*_C_ 3.48/55.2) were observed in the ^1^H and ^13^C NMR spectra. The methine group was assigned to C-2, supported by COSY correlations from H-2 to H-3 and the exchangeable proton 1’-NH, as well as HMBC correlation from 1-NH to C-2 (**Fig. S2)**. Thus, the planar structure of **2** was established as shown in **Fig. 1** in main text and named epicoccin V.

Additionally, the chemical structures of compound **1** and **2** were further confirmed by comparing the NMR data (in MeOH-*d*_4_) with our previous published molecule spiroepicoccin A.(3)

**Fig. S2** Key COSY and HMBC correlations of compounds **1**–**2**.

**Table S1** ^13^C and ^1^H NMR data for compounds **1** and **2** in DMSO-*d*_6_.

| No. | 1 | | 2 | |
| --- | --- | --- | --- | --- |
|  | *δ*_C_^a^ | *δ*_H_^b^ (*J* in Hz) | *δ*_C_^a^ | *δ*_H_^b^ (*J* in Hz) |
| 1 | 164.0, C |  | 166.1, C |  |
| 2 | 91.5, C |  | 55.2, CH | 3.48, td (5.0, 2.1) |
| 3 | 40.1, CH_2_ | *α* 3.42, d (16.4) | 37.4, CH_2_ | *α* 3.12, dd (13.5, 5.0) |
|  |  | *β* 2.90, d (16.4) |  | *β* 2.79, dd (13.5, 5.2) |
| 4 | 125.3, C |  | 136.1, C |  |
| 5 | 120.9, CH | 7.13, d (7.6) | 130.1, CH | 7.14, d (7.6) |
| 6 | 124.2, CH | 6.86, t (7.6) | 128.0, CH | 7.24, t (7.6) |
| 7 | 127.8, CH | 7.10, t (7.6) | 126.5, CH | 7.18, t (7.6) |
| 8 | 108.6, CH | 6.74, d (7.6) | 128.0, CH | 7.24, t (7.6) |
| 9 | 157.0, CH |  | 130.1, CH | 7.14, d (7.6) |
| 1' | 165.8, C |  | 165.4, C |  |
| 2' | 67.0, C |  | 68.0, C |  |
| 3' | 37.4, CH_2_ | *α* 3.40, d (15.2) | 37.9, CH_2_ | *α* 3.21, d (13.6) |
|  |  | *β* 3.19, d (15.2) |  | *β* 3.02, d (13.6) |
| 4' | 121.3, C |  | 121.4, C |  |
| 5' | 155.6, C |  | 155.7, C |  |
| 6' | 115.3, CH | 6.82, d (7.6) | 115.4, CH | 6.78, d (7.6) |
| 7' | 128.2, CH | 7.08, t (7.6) | 128.3, CH | 7.06, t (7.6) |
| 8' | 118.8, CH | 6.72, t (7.6) | 118.8, CH | 6.70, t (7.6) |
| 9' | 130.9, CH | 7.04, d (7.6) | 131.4, CH | 7.05, d (7.6) |
| 2′-SMe | 13.0, CH_3_ | 2.27, s | 11.6, CH_3_ | 1.47, s |
| 5′-OH |  | 9.58, s |  | 9.49, s |
| 1-NH |  | 8.66, s |  | 8.12, s |
| 1′-NH |  | 9.58, s |  | 8.35, d (2.1) |
| *^a^* Data collected at 125 MHz in DMSO-*d*_6_. *^b^* Data collected at 500 MHz in DMSO-*d*_6_. | | | | |

**1.3 Measurement of experimental anisotropic NMR data for spiroepicoccin B (1) and epicoccin V (2)**

We employed the same experimental procedures to measure anisotropic NMR data for compounds **1** and **2** as was previously applied to spiroepicoccin A(3), using the oligopeptide AAKLVFF (4) as the alignment medium. For each of compound **1** and **2**, 1.0 mg of the sample was dissolved in 250 *μ*L MeOH-*d*_4_ and transferred into a 3 mm NMR tube. Afterwards, 1D standard ^13^C and 2D [^1^H, ^13^C]-CLIP-HSQC spectra (5) were collected, respectively. To establish the anisotropic environment, 7.2 mg of AAKLVFF were added into each solution. After shaking the solution vigorously once, the acquisitions of 1D standard ^13^C and 2D [^1^H, ^13^C]-CLIP-HSQC spectra were repeated, allowing for the collection of data under initial alignment state. The samples were then stored at 4 °C and shaken up and down eight times per day until the quadrupolar splitting of the ^2^H signals reached its equilibrium, which were 19.5 and 8.8 Hz (**Fig. S3**) for the OD signal of MeOH-*d*_4_ in the samples of compounds **1** and **2**, respectively. ^13^C and 2D [^1^H, ^13^C]-CLIP-HSQC spectra were acquired a third time to obtain the spectra at the equilibrated alignment condition. Upon analyzing the spectra under isotropic, initial, and equilibrated alignment conditions, 11 RDCs and 18 ∆∆RCSAs for compound **1** (**Fig. S4**, **Table S2**) and 15 RDCs and 19 ∆∆RCSAs for compound **2** (**Fig. S5**, **Table S3**) were extracted with sufficient accuracy.


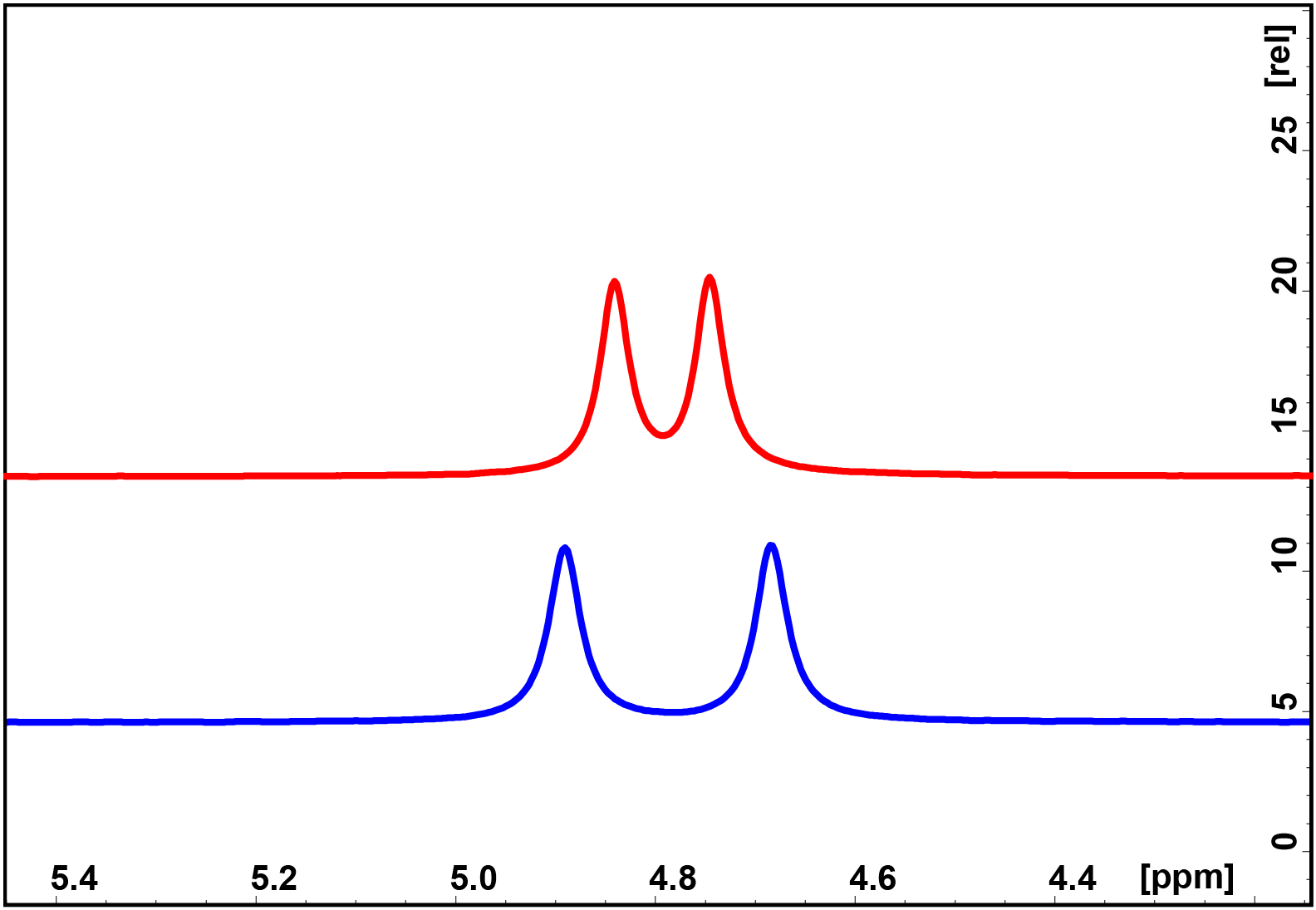


**Fig. S3** The ^2^H splitting of the OD signal of MeOH-*d*_4_ for compound **1** (blue, 19.5 Hz) and **2** (red, 8.8 Hz) in the equilibrated anisotropic phase formed by 28.8 mg/mL AAKLVFF.

**Fig. S4** The 2D chemical structure of compound **1** and the atom numbering (right) used in the DFT NMR calculation at B3LYP/6-311+G (2d,p) level of theory using the implicit solvent model IEFPCM for methanol.

**Table S2** Experimental 11 RDC and 18 ∆∆RCSA data of compound **1**.

| Position | Atom number*^a^* | RDC | ∆∆RCSA*^b^* | ∆∆RCSA*^c^* | ∆∆RCSA*^d^* |
| --- | --- | --- | --- | --- | --- |
| 1 | 18 | – | 0.069 | 0.053 | 0.065 |
| 2 | 8 | – | 0.047 | 0.032 | 0.043 |
| 3 | 9-30 | -21.77 | 0.034 | 0.018 | 0.030 |
|  | 9-31 | -34.01 |  |  |  |
| 4 | 10 | – | -0.112 | -0.128 | -0.115 |
| 5 | 15-35 | 35.04 | -0.145 | -0.160 | -0.149 |
| 6 | 14-34 | 32.19 | -0.142 | -0.161 | -0.145 |
| 7 | 13-33 | 34.17 | -0.145 | -0.152 | -0.146 |
| 8 | 12-32 | 34.16 | -0.125 | -0.141 | -0.130 |
| 9 | 11 | – | -0.091 | -0.107 | -0.093 |
| 1' | 16 | – | 0.045 | 0.030 | 0.041 |
| 2' | 17 | – | 0.000 | -0.015 | 0.004 |
| 3' | 19-(36/37) | – | 0.028 | 0.012 | 0.024 |
| 4' | 20 | – | 0.023 | 0.007 | 0.018 |
| 5' | 21 | – | 0.073 | 0.057 | 0.068 |
| 6' | 22 | -40.19 | 0.083 | 0.064 | 0.078 |
| 7' | 23 | 34.01 | 0.010 | -0.006 | 0.005 |
| 8' | 24 | -29.02 | – | – | – |
| 9' | 25 | -41.68 | 0.093 | 0.081 | 0.092 |
| 2′-*S*Me | 26-(42/43/44) | -5.62 | 0.015 | 0.000 | 0.012 |
| *^a^* Used in the DFT NMR computation at B3LYP/6-311+G(2d,p) level of theory with the implicit solvation in methanol using the IEFPCM model.  *^b,c,d^* Using the carbon atoms C-2', *S*-C and MeOH-*d*_4_, respectively, as reference to extract experimental ∆∆RCSAs. | | | | | |

**Fig. S5** The 2D chemical structure of compound **2** and its atom numbers (right) obtained from DFT NMR calculation under level B3LYP/6-311+G (2d,p) with implicit solvent model IEFPCM for methanol.

**Table S3** Experimental 14 RDC and 19 ∆∆RCSA data of compound **2**.

| Position | Atom number*^a^* | RDC | ∆∆RCSA*^b^* | ∆∆RCSA*^c^* | ∆∆RCSA*^d^* | ∆∆RCSA*^e^* |
| --- | --- | --- | --- | --- | --- | --- |
| 1 | 17 | – | 0.0255 | 0.0202 | 0.0209 | 0.0237 |
| 2 | 7 | 8.16 | 0 | -0.0054 | -0.0047 | -0.0022 |
| 3 | 8-30 | -1.58 | 0.0052 | 0.0000 | 0.0003 | 0.0030 |
|  | 8-31 | -8.91 |  |  |  |  |
| 4 | 9 | – | -0.0111 | -0.0160 | -0.0156 | -0.0132 |
| 5 | 10-32 | -3.09 | 0 | -0.0058 | -0.0056 | -0.0028 |
| 6 | 11-33 | -3.02 | 0 | -0.0058 | -0.0048 | -0.0023 |
| 7 | 12-34 | 7.90 | -0.0127 | -0.0177 | -0.0172 | -0.0146 |
| 8 | 13-35 | -3.02 | 0 | -0.0058 | -0.0048 | -0.0023 |
| 9 | 14-36 | -3.09 | 0 | -0.0058 | -0.0056 | -0.0028 |
| 1' | 15 | – | 0.0240 | 0.0187 | 0.0191 | 0.0215 |
| 2' | 16 | – | 0.0015 | -0.0037 | -0.0032 | -0.0007 |
| 3' | 18-(37/38) | -6.12 | 0.0082 | -0.0027 | 0.0035 | 0.0056 |
| 4' | 19 | – | 0.0010 | -0.0044 | -0.0038 | -0.0013 |
| 5' | 20 | – | 0.0091 | 0.0038 | 0.0045 | 0.0072 |
| 6' | 21 | -6.99 | 0.0136 | 0.0076 | 0.0084 | 0.0115 |
| 7' | 22 | 10.25 | -0.0029 | -0.0081 | -0.0076 | -0.0053 |
| 8' | 23 | -4.45 | 0.0119 | 0.0070 | 0.0074 | 0.0103 |
| 9' | 24 | -6.79 | 0.0119 | 0.0068 | 0.0075 | 0.0095 |
| 2′-*S*Me | 25-(43/44/45) | -1.76 | 0.0048 | -0.0003 | 0.0000 | 0.0029 |
| *^a^* Used in the DFT NMR computation at B3LYP/6-311+G(2d,p) level of theory with the implicit solvation in methanol using the IEFPCM model.  *^b,c,d,e^* Using the carbon atoms C-2, C-3, *S*-C and MeOH-*d*_4_, respectively, as reference to extract experimental ∆∆RCSAs. | | | | | | |

**1.4 Computational methods**

**1.4.1 Computational methods for compound 1**

Relying on the previous strategy developed in our group, first, an initial conformational search was performed using the MacroModel engine in the Maestro V11.4 software suite (Schrödinger Inc.) (6) for two configurations (2*R**, 2’*R** and 2*R**, 2’*S**) of compound **1** using the MMFF94 force field with the implicit Generalized Born/Solvent accessible surface area (GB/SA) solvent model for octanol. Since methanol is not available in Maestro/MacroModel, we chose octanol, as its dielectric constant (epsilon) is the closest to that of methanol among the available solvent models. Generated conformers were kept within an energy window of 10 kJ/mol. Within this window, 10 and 4 possible conformers were found for configurations 2*R**, 2'*R** and 2*R**, 2'*S**, respectively (**Fig. S6**).


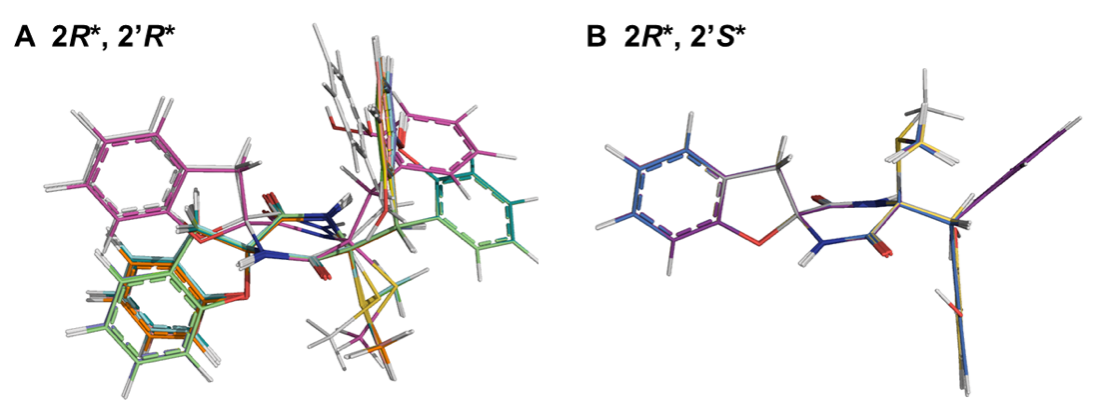


**Fig. S6** Conformational ensembles generated for configurations 2*R**, 2'*R** (10 conformers) and 2*R**, 2'*S** (4 conformers) of compound **1** using MacroModel in Maestro with an energy window of 10 kJ/mol and employing the MMFF94 force field.

Furthermore, density functional theory (DFT)-based structural optimization and frequency calculations were performed in Gaussian16 (7) on the aforementioned 14 conformers at the B3LYP/6-31+G(d,p) level of theory, with implicit solvation in methanol using the IEFPCM model (8). A previous study has suggested that the 6-31+G(d,p) basis set was insufficient for describing sulfur-containing molecules (9). However, as this study also tried larger basis sets, e.g., def2-TZVP, and did not find large differences in the carbon-sulfur bonds, we do not expect our results to be limited by the smaller basis set on this aspect.

Conformer selection was based on the calculation of Boltzmann populations using the difference in free energies ($\Delta G)$ relative to the lowest energy conformer, as given in the following equation:

$$Boltzmann population=\frac{℮^{\frac{-\Delta G}{RT}}}{\sum_{i}^{n} ℮^{\frac{-\Delta G}{RT}}} ,$$

where *R* is the ideal gas constant, *T* is the temperature (298.15 K) and *n* is the number of conformations. All conformers with a Boltzmann probability higher than 5% of the whole ensemble were selected.

Finally, for compound **1**, conformers 8 and 9 were selected for the configuration 2*R**, 2'*R** (*RR*2 in **Fig. 2** in main text) and conformers 1 and 4 for the configuration 2*R**, 2'*S**, as each conformer in these ensembles exhibit a Boltzmann population above 5% (**Table S4)**. These conformers were then used to calculate chemical shielding tensors by DFT employing the gauge-independent atomic orbitals (GIAO) method (10) and subsequently used as structural models for fitting against anisotropic NMR data using the MSpin software (11). The selection of conformational ensembles were also performed by calculating the Boltzmann distribution, using the sum of electronic energy and zero-point energy instead of the Gibbs free energy. For both methods, the ensemble selected for the configuration 2*R**, 2'*S** was the same, while for the configuration 2*R**, 2'*R**, conformers 1, 2, 8 and 9 (*RR*1 in **Fig. 2** in main text) were selected by the latter method, as shown in **Table S5**.

**Table S4** The Boltzmann population of each conformer based on the DFT calculated sum of electronic and thermal free energies for compound **1**.

| Conformer | Sum of electronic and thermal free energies  in [Hartree] | Boltzmann population  in [%] |
| --- | --- | --- |
| **2*R*, 2’*R*** |  |  |
| 1 | -1543.261381 | 1.37 |
| 2 | -1543.260814 | 0.75 |
| 3 | -1543.257566 | 0.02 |
| 4 | -1543.260057 | 0.34 |
| 5 | -1543.259736 | 0.24 |
| 6 | -1543.259790 | 0.26 |
| 7 | -1543.257537 | 0.02 |
| 8 | -1543.264129 | 24.8 |
| 9 | -1543.265141 | 71.9 |
| 10 | -1543.259975 | 0.31 |
|  |  |  |
| **2*R*, 2’*S*** |  |  |
| 1 | -1543.263821 | 79.94 |
| 2 | -1543.257021 | 0.06 |
| 3 | -1543.258390 | 0.26 |
| 4 | -1543.262493 | 19.74 |

**Table S5** The Boltzmann population of each conformer based on the DFT calculated sum of electronic and zero-point energies for compound **1**.

| Conformer | Sum of electronic and zero-point energies  in [Hartree] | Boltzmann population  in [%] |
| --- | --- | --- |
| **2*R*, 2’*R*** |  |  |
| 1 | -1543.213466 | 17.5 |
| 2 | -1543.212985 | 10.5 |
| 3 | -1543.210018 | 0.50 |
| 4 | -1543.209593 | 0.30 |
| 5 | -1543.211935 | 3.50 |
| 6 | -1543.211853 | 3.20 |
| 7 | -1543.207628 | 0.00 |
| 8 | -1543.214162 | 36.3 |
| 9 | -1543.213909 | 27.8 |
| 10 | -1543.209894 | 0.40 |
|  |  |  |
| **2*R*, 2’*S*** |  |  |
| 1 | -1543.212525 | 34.1 |
| 2 | -1543.209614 | 1.80 |
| 3 | -1543.210310 | 3.30 |
| 4 | -1543.213057 | 60.8 |

**1.4.2 Computational methods for compound 2**

Considering the priori higher flexibility of compound **2** compared to compound **1**, a larger energy window of 30 kJ/mol was chosen for the conformational search in MacroModel. This decision was based on a previous study showing that increasing the energy window in CASE-3D calculations allows for a more exhaustive search of possible conformers (12), while another publication indicating that the MMFF force field can exhibit an energy error of up to 20 kJ/mol (13).

As a result, 25 and 8 conformers were generated for the two possible configurations 2*R**, 2'*R** and 2*R**, 2'*S**, respectively (**Fig. S7**). The same DFT optimization and NMR calculation were then performed for compound **2** as were done for **1**. Finally, six conformers — 2, 3, 7, 15, 22 and 24 for the configuration 2*R**, 2'*R**, and two conformers — 1 and 8 for the configuration 2*R**, 2'*S* were selected based on their Boltzmann populations (**Table S6**), using the sum of electronic and thermal free energies. For the RDC and RCSA analyses in MSpin for the configuration 2*R**, 2'*R*, only four conformers 2, 3, 15 and 22 were used as shown in **Fig. 3** in the main text, as the conformers 3 and 7 and 15 and 24 have identical structures and show similar Gibbs free energies.

Due to the higher conformational flexibility of compound **2** compared to the compound **1**, the above-described DFT calculations may not adequately account for long-range intramolecular interactions. Consequently, we repeated the structural optimizations and frequency calculations for all 32 conformers of compound **2**, incorporating empirical dispersion correction using the B3LYP-D3(BJ)/6-31+G(d,p) level of theory (14-17) and the same IEFPCM (methanol) solvation model. The inclusion of dispersion correction by D3(BJ) significantly influenced the geometries and free energies of the conformers. A comparison between the ensembles and their populations obtained from the B3LYP and B3LYP-D3(BJ) computations is given in Table **S8**. As a result, different structural ensembles were selected based on the Boltzmann populations (**Table S7)** for 2*R**, 2'*R** (6 conformers: 2, 3, 8, 15, 17 and 22) and 2*R**, 2'*S** (4 conformers: 1, 2, 4 and 8), as depicted in **Fig. 4A** in the main text.

All of the above mentioned DFT calculations were performed with Gaussian16. The parameters applied in each DFT calculations are as follows:

(A) DFT optimizations and free energy calculations:

# opt freq b3lyp/6-31+g(d,p) scrf=(iefpcm, solvent=methanol) geom=connectivity

(B) DFT calculation of chemical shielding tensors:

# nmr=giao b3lyp/6-311+g(2d, p) scrf=(iefpcm, solvent=methanol) geom=connectivity

(C) DFT optimizations and free energy calculations with empirical dispersion correction:

# opt freq b3lyp/6-31+g(d,p) scrf=(iefpcm, solvent=methanol) geom=connectivity, EmpiricalDispersion=GD3BJ

(D) DFT calculation of chemical shielding tensors with empirical dispersion correction:

# nmr=giao b3lyp/6-311+g(2d, p) scrf=(iefpcm, solvent=methanol) geom=connectivity EmpiricalDispersion=GD3BJ

**
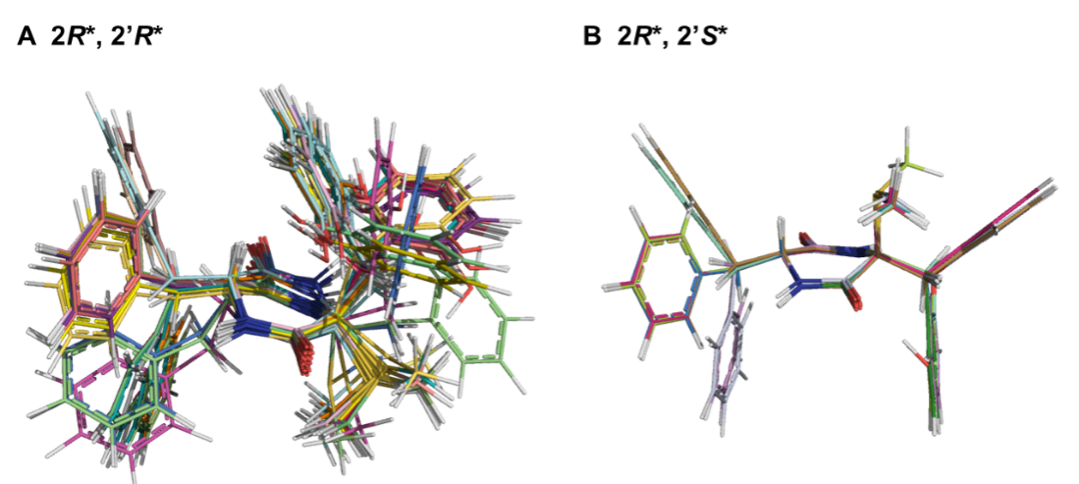
**

**Fig. S7** Structural ensembles generated for configurations 2*R**, 2'*R** (25 conformers) and 2*R**, 2'*S** (10 conformers) of compound **2** using MacroModel in Maestro with an energy window of 30 kJ /mol and employing the MMFF94 force field.

**Table S6** The Boltzmann populations based on the DFT calculated sum of electronic and thermal free energies for compound **2** at B3LYP/6-31+G (d,p) level of theory with implicit IEFPCM solvation in methanol.

| Conformer | Sum of electronic and thermal free energies  in [Hartree] | Boltzmann  populations  in [%] |
| --- | --- | --- |
| **2*R*, 2’*R*** |  |  |
| CS1 | -1469.252321 | 0.20 |
| CS2 | -1469.254775 | 6.20 |
| CS3 | -1469.255952 | 19.6 |
| CS4 | -1469.252131 | 0.20 |
| CS5 | -1469.252950 | 0.50 |
| CS6 | -1469.251433 | 0.10 |
| CS7 | -1469.255954 | 19.8 |
| CS8 | -1469.251833 | 0.30 |
| CS9 | -1469.253414 | 0.80 |
| CS10 | -1469.249168 | 0.00 |
| CS11 | -1469.249535 | 0.00 |
| CS12 | -1469.251816 | 0.30 |
| CS13 | -1469.251499 | 0.10 |
| CS14 | -1469.247654 | 0.00 |
| CS15 | -1469. 254767 | 5.70 |
| CS16 | -1469.250371 | 0.00 |
| CS17 | -1469.251822 | 0.60 |
| CS18 | -1469.249441 | 0.00 |
| CS19 | -1469.248432 | 0.00 |
| CS20 | -1469.251580 | 0.10 |
| CS21 | -1469.250074 | 0.00 |
| CS22 | -1469.256385 | 41.9 |
| CS23 | -1469.248146 | 0.00 |
| CS24 | -1469.254767 | 5.70 |
| CS25 | -1469.247312 | 0.00 |
| **2*R*, 2’*S*** |  |  |
| CS1 | -1469.256623 | 14.0 |
| CS2 | -1469.253850 | 1.00 |
| CS3 | -1469.255215 | 2.00 |
| CS4 | -1469.253367 | 0.60 |
| CS5 | -1469.250619 | 0.10 |
| CS6 | -1469.252354 | 0.20 |
| CS7 | -1469.249916 | 0.00 |
| CS8 | -1469.257422 | 82.0 |

**Table S7** The Boltzmann populations based on the DFT calculated sum of electronic and thermal free energies for compound **2** at B3LYP-D3(BJ)/6-31+G (d,p) level of theory with implicit IEFPCM solvation in methanol.

| Conformer | Sum of electronic and thermal free energies  in [Hartree] | Boltzmann  population  in [%] |
| --- | --- | --- |
| **2*R*, 2’*R*** |  |  |
| CS1 | -1469.358458 | 0.07 |
| CS2 | -1469.363025 | 9.15 |
| CS3 | -1469.363178 | 10.8 |
| CS4 | -1469.360149 | 0.44 |
| CS5 | -1469.361315 | 1.51 |
| CS6 | -1469.357301 | 0.02 |
| CS7 | -1469.359861 | 0.33 |
| CS8 | -1469.364061 | 27.3 |
| CS9 | -1469.358991 | 0.13 |
| CS10 | -1469.361021 | 1.11 |
| CS11 | -1469.359608 | 0.25 |
| CS12 | -1469.359230 | 0.17 |
| CS13 | -1469.359271 | 0.18 |
| CS14 | -1469.356818 | 0.01 |
| CS15 | -1469.362404 | 4.76 |
| CS16 | -1469.355827 | 0.00 |
| CS17 | -1469.362445 | 4.97 |
| CS18 | -1469.359195 | 0.16 |
| CS19 | -1469.354041 | 0.00 |
| CS20 | -1469.358106 | 0.05 |
| CS21 | -1469.357732 | 0.03 |
| CS22 | -1469.364389 | 38.5 |
| CS23 | -1469.356082 | 0.01 |
| CS24 | -1469.359113 | 0.15 |
| CS25 | -1469.356651 | 0.01 |
| **2*R*, 2’*S*** |  |  |
| CS1 | -1469.362693 | 12.6 |
| CS2 | -1469.362228 | 7.74 |
| CS3 | -1469.361386 | 3.19 |
| CS4 | -1469.362816 | 14.4 |
| CS5 | -1469.35904 | 0.2 |
| CS6 | -1469.358698 | 0.19 |
| CS7 | -1469.357188 | 0.04 |
| CS8 | -1469.364197 | 61.6 |

**Table S8** Comparison between the ensemble obtained from the free energy calculation using the structures optimized with B3LYP and B3LYP-D3(BJ) and their subsequent populations in the ensembles determined by the RDC+RCSA analysis^a^.

| Conformer | B3LYP | | | B3LYP-D3(BJ) | | |
| --- | --- | --- | --- | --- | --- | --- |
|  | | Population based on Free energy [%] | Population based on RDC+RCSA^a^ [%] | Population based on Free energy [%] | Population based on RDC+RCSA^a^ [%] | |
| **2*R*, 2’*R*** |  | |  |  | |  |
| CS2 | 6 | | 0 | 9 | | 17 |
| CS3^b^ | 43 | | 0 | 11 | | 0 |
| CS8 | 0 | | ^d^ | 27 | | 38 |
| CS15^c^ | 6 | | 55 | 5 | | 0 |
| CS17 | 1 | | ^d^ | 5 | | 45 |
| CS22 | 34 | | 45 | 39 | | 0 |
| **2*R*, 2’*S*** |  | |  |  | |  |
| CS1 | 14 | | 31 | 13 | | 14 |
| CS2 | 1 | | ^d^ | 8 | | 38 |
| CS4 | 1 | | ^d^ | 14 | | 42 |
| CS8 | 82 | | 69 | 62 | | 6 |

^a^ Ensembles from the RDC+RCSA analysis with *S*-C as reference.  ^b^ Sum of Boltzmann population of CS3 and CS7 as they have the same structure. ^c^ Sum of Boltzmann population of CS15 and CS24 as they have the same structure. ^d^ Was not used in the RDC+RCSA analysis due to populations smaller than 5%.

Both CREST (**C**onformer-**R**otamer **E**nsemble **S**ampling **T**ool) and CENSO (**C**ommandline **EN**ergetic **SO**rting of Conformer Rotamer Ensembles) have been recently introduced by Grimme *et al*. (18, 19). These techniques offer an alternative approach to conformational sampling compared to MacroModel in Maestro. To explore the difference between these two sampling approaches, we conducted a conformational search for compound **2** using CREST at the GFN2-xTB level of theory (20, 21). The unique conformers identified within a 6 kcal/mol energy window underwent further conformational refinement and optimization using the CENSO software (19). For the prescreening and subsequent DFT optimization, B3LYP-D4/def2-TZVP(-f) and SMD implicit solvation (22) with parameters for methanol were applied. Finally, based on the Gibbs free energy, calculated by employing the modified rigid-rotor harmonic oscillator approximation (mRRHO) (23) based on GFN2-xTB at 298.15 K, the DFT optimized ensembles for the two possible configurations 2*R**, 2'*R** (conformers 3, 9, 28 and 40) and 2*R**, 2'*S** (conformers 1,3, 4 and 5) of compound **2** were obtained with Boltzmann populations above 5% (**Table S9** and **Fig. 4B** in main text**)**. The following commands have been used for CREST and CENSO.

CREST: Conformational search using GFN2-xTB with implicit solvation using ALPB in methanol

- crest input_geometry.xyz --gfn2 --alpb methanol -nmr

CENSO: Ensemble optimization and GIAO-DFT calculations using B3LYP-D4(def2-TZVP) and SMD solvation in methanol.

- censo --input crest_conformers.xyz -func b3lyp-d4 -func0 -b3lyp-d4 -solvent methanol -- smgsolv1 smd -sm2 smd --smgsolv2 smd --prog orca -part4 on -prog4J orca -prog4S orca -basisJ def2-TZVP -basisS def2-TZVP -cactive on

**Table S9** The Boltzmann populations based on the DFT calculated Gibbs free energy in CENSO for compound **2** at B3LYP-D4/def2-TZVP(-f) level of theory with implicit SMD solvation in methanol.

| Conformer | Gibbs free energy  in [Hartree] | Boltzmann  populations  in [%] |
| --- | --- | --- |
| **2*R*, 2’*R*** |  |  |
| CS2 | -1469.03341 | 1.36 |
| CS3 | -1469.03665 | 42.2 |
| CS4 | -1469.03322 | 1.12 |
| CS5 | -1469.03418 | 3.07 |
| CS8 | -1469.03397 | 2.46 |
| CS9 | -1469.03609 | 23.2 |
| CS15 | -1469.03419 | 3.10 |
| CS16 | -1469.03361 | 1.68 |
| CS28 | -1469.03495 | 6.95 |
| CS40 | -1469.03474 | 5.59 |
| CS42 | -1469.03433 | 3.60 |
| CS47 | -1469.03346 | 1.44 |
| CS48 | -1469.03271 | 0.65 |
| CS49 | -1469.03420 | 3.15 |
| CS71 | -1469.03241 | 0.47 |
| **2*R*, 2’*S*** |  |  |
| CS1 | -1469.03616 | 9.53 |
| CS2 | -1469.03458 | 1.78 |
| CS3 | -1469.03782 | 55.1 |
| CS4 | -1469.03649 | 13.4 |
| CS5 | -1469.03611 | 9.00 |
| CS8 | -1469.03500 | 2.77 |
| CS11 | -1469.03366 | 0.67 |
| CS13 | -1469.03348 | 0.56 |
| CS23 | -1469.03462 | 1.87 |
| CS30 | -1469.03444 | 1.54 |
| CS42 | -1469.03453 | 1.70 |
| CS68 | -1469.03470 | 2.02 |

In CASE-3D, the best ensemble is selected by calculating the Akaike information criterion (*AIC*) for all provided structural models. The *AIC* value for a model is determined by taking both experimental and theoretical data into account (24, 25) by comparing the *AIC* values or conformational ensembles belonging to different configurations. Among all structural models, those with the lowest *AIC*s are generally recognized as the correct configuration and the best conformational ensemble.

With the acquisition of standard ^1^H and ^13^C NMR experiments in MeOH-*d*_4_, 11 ^1^H chemical shifts and 19 ^13^C chemical shifts were extracted for compound **2**, as shown in **Table 1** of the main text. In Stereofitter as implemented in the MSpin software, the DFT computed isotropic shielding constants were transformed into chemical shifts using the following linear relationship, making them comparable with the experimental shifts:

$$\delta_{13C}=-0.923\sigma_{13C}^{DFT}+169.0$$

$$\delta_{1H}=-0.918\sigma_{1H}^{DFT}+29.36$$

To account for the heavy-atom effect of the sulfur atom over the ^13^C shift a correction of +5 ppm was added to the experimental shifts of the sulfur-attached carbon atoms in the StereoFitter files.

**1.5 Determination of relative configuration for compound 1**

For compound **1**, in total 11 RDCs and 18 ^13^C ∆∆RCSAs were collected with sufficient accuracy. Three different carbon atoms were selected as references for the extraction of the ∆∆RCSAs: (ⅰ) the carbon atom with the smallest theoretically calculated chemical shift anisotropy (CSA) (C-2’); (ⅱ) the only methyl carbon of compound **1** which showed the smallest chemical shift change between initial and equilibrated alignment states (*S*-C); and (ⅲ) the methyl carbon atom of the solvent MeOH-*d*_4_.

The comparison of the inter-tensor angles between the RDC and RCSA derived alignment tensors offers another possibility to evaluate the agreement between the structural models and the experimental data. As RDC and RCSA originate from the same alignment tensor, a theoretical inter-tensor angle of 0° is expected between the alignment tensors derived from their measurements. All inter-tensor angles from the selected structural models, selected by DFT energies of compound **1**, are summarized in **Table S10**. Alignment tensors and back-computed RDCs and RCSAs were obtained using the Singular Value Decomposition (SVD) (26) approach as implemented in MSpin. Conformational amplitudes were determined through simultaneous least-square optimization.

All cartesian coordinates of the structural ensembles, the input files and the output files for the structural elucidation of **1** using 11 RDCs and 18 ∆∆RCSAs in either separate or simultaneous SVD fits in MSpin were deposited on Zenodo named Compound **1_**additional files (doi.org/10.5281/zenodo.10986434).

**Table S10** Inter-tensor angles between RDC and RCSA derived alignment tensors of the conformational ensembles for compound **1** selected by DFT energies. *^a^*

| **Inter-tensor angles for structural models based on DFT energies using the sum of electronic and zero-point energies (Hartree)** | | |
| --- | --- | --- |
|  | 2*R^*^,* 2*R^*^* | 2*R^*^,* 2*S^*^* |
|  | Conformers 1, 2, 8, 9 | Conformers 1, 4 |
|  | RDC | RDC |
| RCSA-C2 | 109.2° | 18.1° |
| RCSA-*S*C | 100.6° | 22.8° |
| RCSA- MeOH-*d*_4_ | 108.2° | 18.0° |
| **Inter-tensor angles for structural models based on DFT energies using the sum of electronic and thermal free energies (Hartree)** | | |
|  | 2*R^*^,* 2*R^*^* | 2*R^*^,* 2*S^*^* |
|  | Conformers 8, 9 | Conformers 1, 4 |
|  | RDC | RDC |
| RCSA-C2 | 11.6° | 36.6° |
| RCSA-*S*C | 34.4° | 22.0° |
| RCSA- MeOH-*d*_4_ | 32.0° | 37.4° |

**1.6 Determination of relative configuration for compound 2**

For compound **2**, 15 RDCs and 19 ^13^C ∆∆RCSAs were extracted with sufficient accuracy. For the extraction of the ∆∆RCSAs, four different carbon atoms were selected as references: (ⅰ) the carbon atom with the smallest theoretically calculated CSA (C-2 and C-3); (ⅱ) the only methyl carbon of compound **2** which showed the smallest chemical shift change between initial and equilibrated alignment states (*S*-C); and (ⅲ) the methyl carbon atom of the solvent MeOH-*d*_4_.

The structural ensembles of compound **2** were selected not only based on DFT free energies (**Fig. 3** and **4A/4B** in main text) but also based on the CASE-3D approach (**Fig. 4E/4G** in main text). Moreover, except for the structural ensembles with the lowest *AIC*s, those with the second lowest *AIC*s found using CASE-3D were also considered and applied in the further RDC and RCSA fitting procedure to determine the relative configuration of compound **2** as shown in **Fig. S8**. The conformational distribution of compound **2** derived from CASE-3D calculations were summarized in **Fig. S9**. Additionally, the resulting chemical shifts for the ensembles with the lowest *AIC* values in CASE-3D are listed in **Table S11** and shown in comparison to the experimental shift data for hydrogen shifts in **Fig. S10** and carbon shifts in **Fig.** **S11**. Regardless of which structural ensembles were employed in further anisotropic NMR data analyses, the conformers show a larger population always exhibit a more folded shape compared to the other selected conformers.

Alignment tensors, along with back-computed RDCs and RCSAs, were calculated using the Singular Value Decomposition (SVD) approach as implemented in MSpin. Conformational amplitudes were determined through simultaneous least-square optimization. All inter-tensor angles from various structural models, selected based on DFT energies and CASE-3D for compound **2**, are summarized in **Table S12-Table S15**.

All cartesian coordinates of the structural ensembles, the input files and the output files for the structural elucidation of **2** using 15 RDCs and 19 ∆∆RCSAs in either separate or simultaneous SVD fits in MSpin were deposited on Zenodo named Compound **2_**additional files (doi.org/10.5281/zenodo.10986434).

**
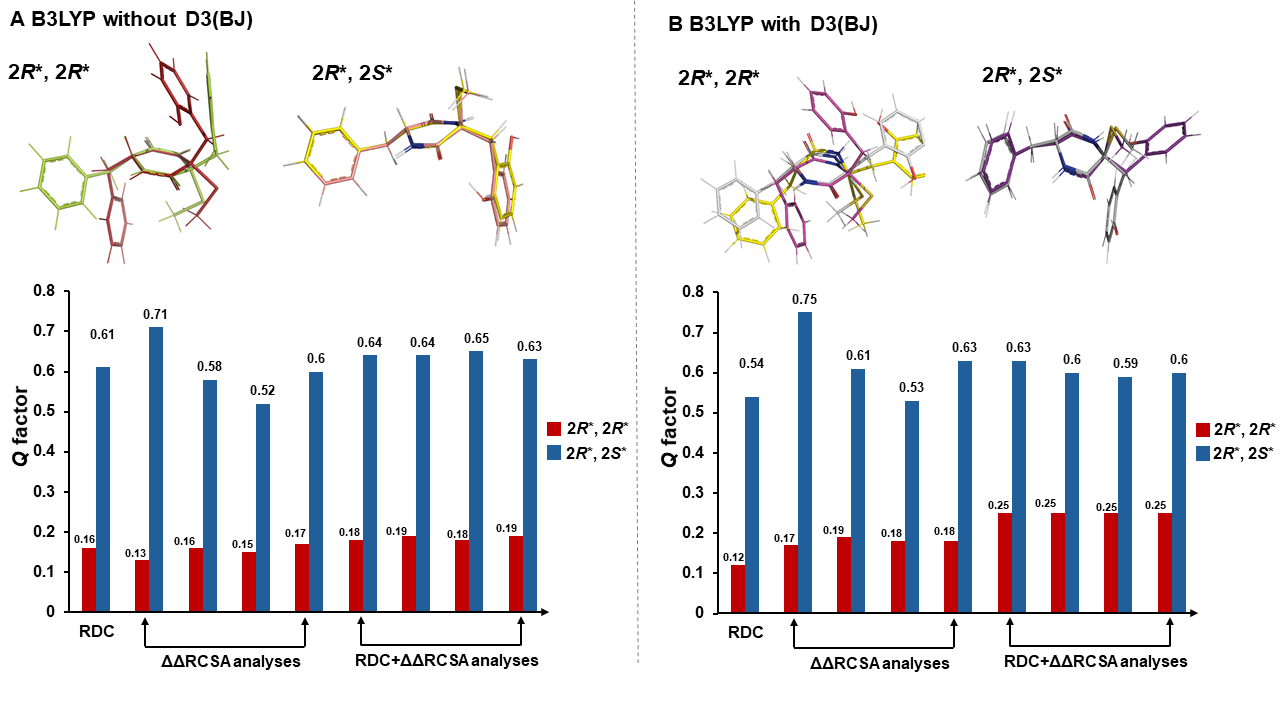
Fig. S8** (**A**) Structural ensembles with the second lowest *AIC* values selected by CASE-3D and the calculated *Q* factors for the RDC and ΔΔRCSA fitting with SVD method using DFT optimized structures at B3LYP/6-31+G(d,p) level of theory. (**B**) Structural ensembles with the second lowest *AIC* values selected by CASE-3D and the calculated *Q*-Factors for the RDC and ΔΔRCSA fitting with SVD method using DFT optimized structures at B3LYP-D3(BJ)/6-31+G(d,p) level of theory. For the ΔΔRCSA analyses, the four reference carbons from left to right are C-2, C-3, S-C and MeOH-d4.

**
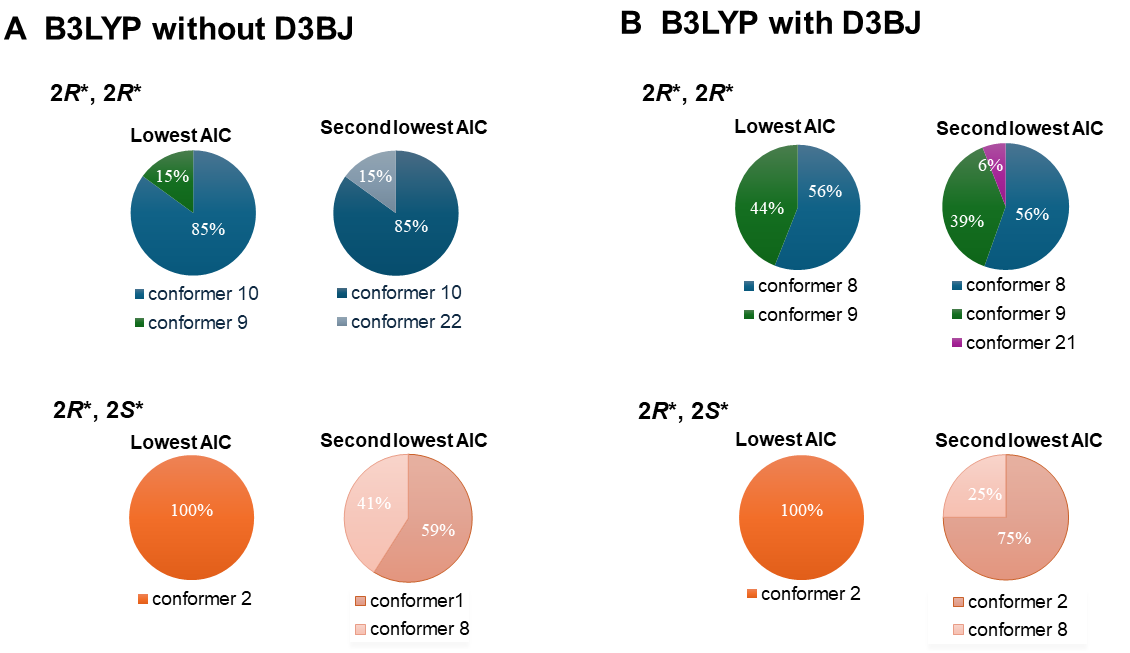
Fig. S9** Structural ensembles and the population of each conformer selected by CASE-3D for configurations 2*R**, 2'*R** and 2*R**, 2'*S** of compound **2** based on DFT optimized conformers at the level (A) B3LYP/6-31+G(d,p) and (B) B3LYP-D3(BJ)/6-31+G(d,p). Both optimizations included implicit solvation in methanol using the IEFPCM model.

**Table S11** The chemical shifts for compound **2** calculated with B3LYP/6-311+G(2d,p) and IEFPCM(methanol) with the structural ensembles determined using CASE-3D with the lowest *AIC* values using the structures optimized at B3LYP/6-31+G(d,p) and B3LYP-D3(BJ)/6-31+G(d,p).

| Atom-Number | Element | Experimental | B3LYP | | B3LYP-D3(BJ) | |
| --- | --- | --- | --- | --- | --- | --- |
|  |  | in [ppm] | *RR*  in [ppm] | *RS*  in [ppm] | *RR*  in [ppm] | *RS*  in [ppm] |
| 17 | C | 168.8 | 162.3 | 164.5 | 163.9 | 164.7 |
| 7 | C | 56.9 | 58.6 | 57.5 | 58.7 | 56.8 |
| 8 | C | 38.9 | 38.8 | 37.8 | 39.4 | 37.0 |
| 9 | C | 136.8 | 134.2 | 135.0 | 133.8 | 134.7 |
| 10,14 | C | 131.5 | 126.8 | 125.7 | 126.5 | 125.3 |
| 11,13 | C | 129.4 | 124.6 | 125.4 | 124.8 | 125.5 |
| 12 | C | 128.2 | 123.3 | 123.5 | 123.5 | 123.5 |
| 15 | C | 168.5 | 160.5 | 160.4 | 161.7 | 160.4 |
| 16 | C | 75.7 | 75.8 | 73.5 | 74.3 | 72.5 |
| 18 | C | 40.1 | 41.4 | 37.2 | 38.6 | 35.3 |
| 19 | C | 122.2 | 118.1 | 120.3 | 119.0 | 120.0 |
| 20 | C | 157.3 | 151.6 | 150.3 | 150.5 | 149.6 |
| 21 | C | 116.4 | 111.1 | 111.1 | 110.9 | 110.9 |
| 22 | C | 130.1 | 125.5 | 125.6 | 125.5 | 125.3 |
| 23 | C | 120.6 | 116.3 | 117.3 | 116.6 | 117.0 |
| 24 | C | 133 | 130.8 | 130.3 | 128.8 | 129.8 |
| 25 | C | 17.1 | 17.3 | 20.1 | 18.1 | 19.7 |
| 26 | H | 3.39 | 3.5 | 4.2 | 3.4 | 4.2 |
| 30,31 | H | 3.01 | 3.0 | 3.1 | 3.1 | 3.0 |
| 32,36 | H | 7.16 | 7.1 | 7.2 | 7.1 | 7.2 |
| 33,35 | H | 7.23 | 7.2 | 7.3 | 7.2 | 7.3 |
| 34 | H | 7.2 | 7.1 | 7.2 | 7.1 | 7.2 |
| 39 | H | 6.79 | 6.6 | 6.7 | 6.6 | 6.7 |
| 40 | H | 7.11 | 7.0 | 7.1 | 7.1 | 7.1 |
| 41 | H | 6.76 | 6.7 | 6.8 | 6.8 | 6.8 |
| 42 | H | 7.08 | 7.0 | 7.1 | 7.1 | 7.2 |
| 37,38 | H | 3.23 | 3.2 | 3.4 | 3.2 | 3.4 |
| 43,44,45 | H | 1.47 | 1.5 | 2.1 | 1.5 | 2.0 |
| MAE |  |  | 2.2 | 2.3 | 2.3 | 2.5 |


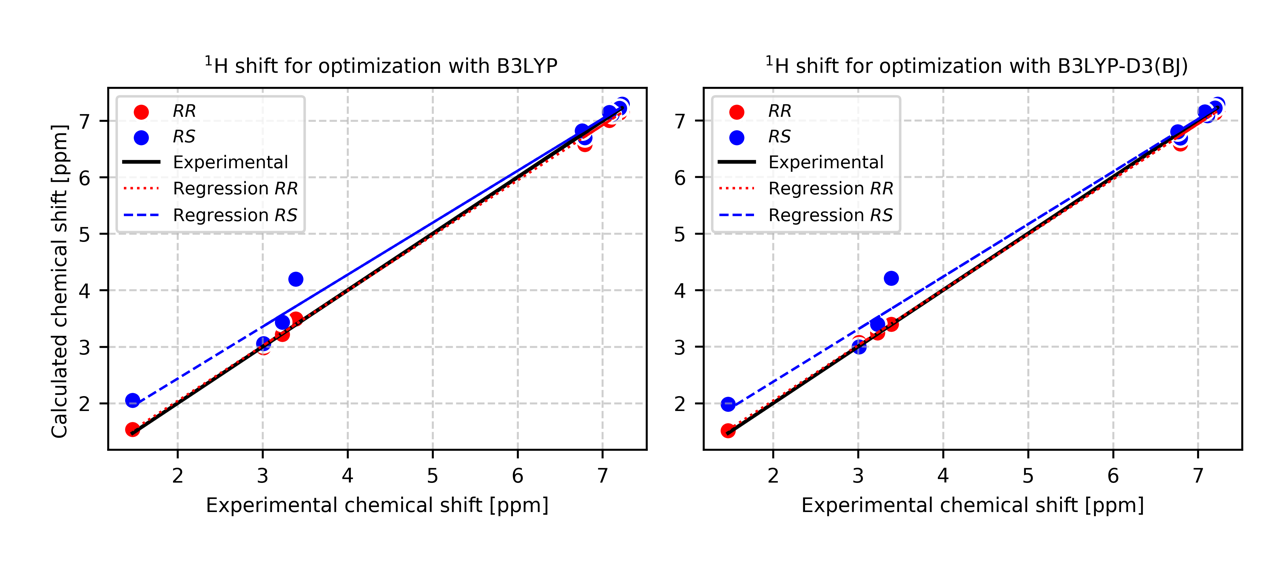


**Fig. S10** Correlation between experimental and calculated proton chemical shifts for the structural ensembles with the lowest *AIC* value by CASE-3D. NMR shielding was calculated using the B3LYP/6-311+G(2d,p) with IEFPCM(methanol) level of theory.


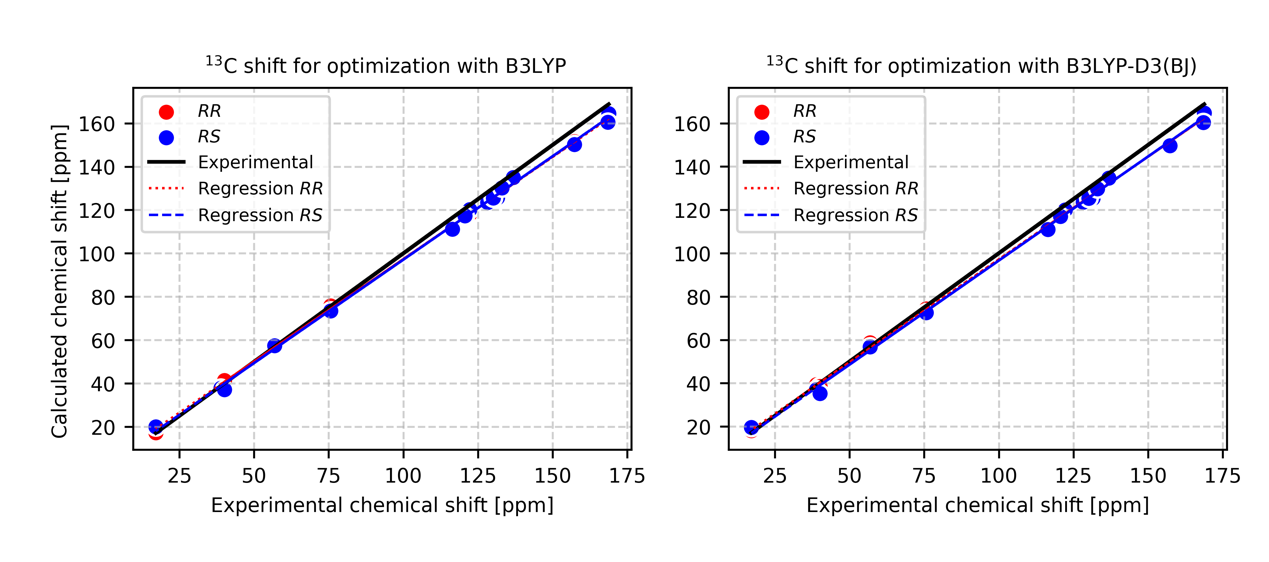


**Fig. S11** Correlation between experimental and calculated carbon chemical shifts for the structural ensembles with the lowest *AIC* value by CASE-3D. NMR shielding was calculated using the B3LYP/6-311+G(2d,p) with IEFPCM(methanol) level of theory.

**Table S12** Inter-tensor angles between the RDC and RCSA derived alignment tensors of compound **2** as obtained from the SVD analysis in MSpin using the structural ensembles selected based on the DFT free energies calculated with D4 empirical dispersion correction.*^a^*

| **Inter-tensor angles for structural models based on DFT energies with D4** | | |
| --- | --- | --- |
|  | 2*R^*^,* 2*R^*^* | 2*R^*^,* 2*S^*^* |
|  | Conformers 3, 9, 28 and 40 | Conformers 1, 3, 4 and 5 |
|  | RDC | RDC |
| RCSA-C2 | 22.9° | 44.8° |
| RCSA-C3 | 25.5° | 32.2° |
| RCSA-*S*C | 26.4° | 105.6° |
| RCSA- MeOH-*d*_4_ | 25.3° | 37.4° |

*^a^* All conformers were generated using the CREST/CENSO approach.

**Table S13** Inter-tensor angles between the RDC and RCSA derived alignment tensors of compound **2** as obtained from the SVD analysis in MSpin using the structural ensembles selected based on the DFT free energies calculated with D3(BJ) empirical dispersion correction. *^a^*

| **Inter-tensor angles for structural models based on DFT energies without D3(BJ)** | | |
| --- | --- | --- |
|  | 2*R^*^,* 2*R^*^* | 2*R^*^,* 2*S^*^* |
|  | Conformers 3, 15 and 22 | Conformers 1 and 8 |
|  | RDC | RDC |
| RCSA-C2 | 73.6° | 14.4° |
| RCSA-C3 | 72.3° | 14.4° |
| RCSA-*S*C | 71.8° | 15.5° |
| RCSA- MeOH-*d*_4_ | 72.3° | 16.1° |
| **Inter-tensor angles for structural models based on DFT energies with D3(BJ)** | | |
|  | 2*R^*^,* 2*R^*^* | 2*R^*^,* 2*S^*^* |
|  | Conformers 2, 3,8,15,17 and 22 | Conformers 1, 2, 4 and 8 |
|  | RDC | RDC |
| RCSA-C2 | 22.1° | 50.7° |
| RCSA-C3 | 97.9° | 108.7° |
| RCSA-*S*C | 30.3° | 108.5° |
| RCSA- MeOH-*d*_4_ | 30.4° | 108.7° |

*^a^* All conformers generated from Maestro.

**Table S14** Inter-tensor angles between the RDC and RCSA derived alignment tensors of compound **2** as obtained from the SVD analysis in MSpin using the structural ensembles with the lowest *AIC*s based on the CASE-3D approach with and without D3(BJ) empirical dispersion correction.*^a^*

| **Inter-tensor angles for structural models from CASE-3D without D3(BJ)** | | |
| --- | --- | --- |
|  | 2*R^*^,* 2*R^*^* | 2*R^*^,* 2*S^*^* |
|  | Conformers 9 and 10 | Conformer 2 |
|  | RDC | RDC |
| RCSA-C2 | 101° | 104.4° |
| RCSA-C3 | 102.4° | 105.2° |
| RCSA-*S*C | 101.8° | 89.9° |
| RCSA- MeOH-*d*_4_ | 102.6° | 106.3° |
| **Inter-tensor angles for structural models from CASE-3D with D3(BJ)** | | |
|  | 2*R^*^,* 2*R^*^* | 2*R^*^,* 2*S^*^* |
|  | Conformers 8 and 9 | Conformer 2 |
|  | RDC | RDC |
| RCSA-C2 | 25.1° | 95.5° |
| RCSA-C3 | 26.5° | 104.2° |
| RCSA-*S*C | 20.2° | 84.4° |
| RCSA- MeOH-*d*_4_ | 25.3° | 104.6° |

*^a^* All conformers generated from Maestro.

**Table S15** Inter-tensor angles between the RDC and RCSA derived alignment tensors of compound **2** as obtained from the SVD analysis in MSpin using the structural ensembles with the second lowest *AIC*s based on the CASE-3D approach with and without D3(BJ) empirical dispersion correction.*^a^*

| **Inter-tensor angles for structural models from CASE-3D without D3(BJ)** | | |
| --- | --- | --- |
|  | 2*R^*^* 2*R^*^* | 2*R^*^* 2*S^*^* |
|  | Conformers 10 and 22 | Conformers1 and 8 |
|  | RDC | RDC |
| RCSA-C2 | 15.7° | 14.4° |
| RCSA-C3 | 22.5° | 14.4° |
| RCSA-*S*C | 29.7° | 15.5° |
| RCSA- MeOH-*d*_4_ | 24.1° | 16.1° |
| **Inter-tensor angles for structural models from CASE-3D with D3(BJ)** | | |
|  | 2*R^*^* 2*R^*^* | 2*R^*^* 2*S^*^* |
|  | Conformers 8, 9 and 21 | Conformers 2 and 8 |
|  | RDC | RDC |
| RCSA-C2 | 70.6° | 83.9° |
| RCSA-C3 | 70.9° | 77.6° |
| RCSA-*S*C | 70.7° | 75.2° |
| RCSA- MeOH-*d*_4_ | 70.7° | 75.4° |

*^a^* All conformers generated from Maestro.

**1.7 DFT calculation of intramolecular methyl-π interaction for compound 2**

The visualization of noncovalent interactions of the conformers CS8 and CS9 optimized with B3LYP-D3(BJ)/6-31+G (d, p) were based on DFT densities of B3LYP-D3(BJ)/6-311+G(2d,p) level of theory obtained with the implicit solvent model SMD (methanol) using ORCA 5.0.4 (27, 28). The atoms-in-molecules (AIM) flag was used in order to generate the density files (wfn and wfx), which were subsequently analyzed using the software Multiwfn 3.7 (29). The *S*-CH_3_ group and the phenyl ring were selected for the analysis using the independent gradient model based on Hirshfeld partition (IGMH) (30). Resulting volumetric data (.cube format) files were visualized as described by the author using VMD. (31) Based on the IGMH framework, the interaction can be classified as a van-der-Waals interaction.

**1.8 Determination of absolute configurations for compound 1 and 2 using TDDFT-ECD calculation (32, 33).**


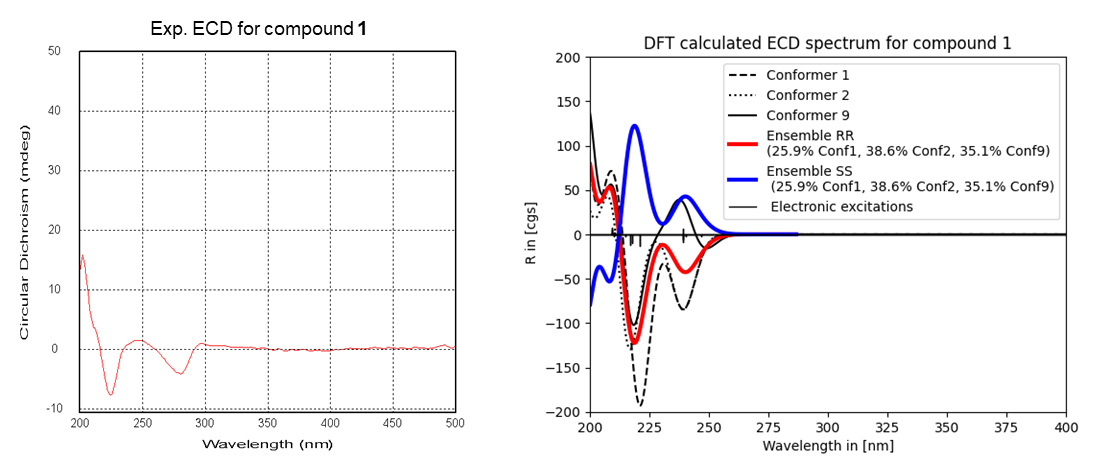


**Fig. S12** Experimental and back-calculated ECD spectrum of compound **1**, which reveals the absolute configuration of compound **1** to be 2*R*, 2'*R*. The ECD spectrum was calculated after reoptimizing the conformers at the DFT level B3LYP-D4/def2-TZVP using the implicit SMD solvation model for methanol. For the TD-DFT calculation the DFT level ωB97-X/def2-TZVPPD (34) in combination with implicit SMD solvation in methanol was used and 50 roots were selected for the calculation of the electronic excitations.

**
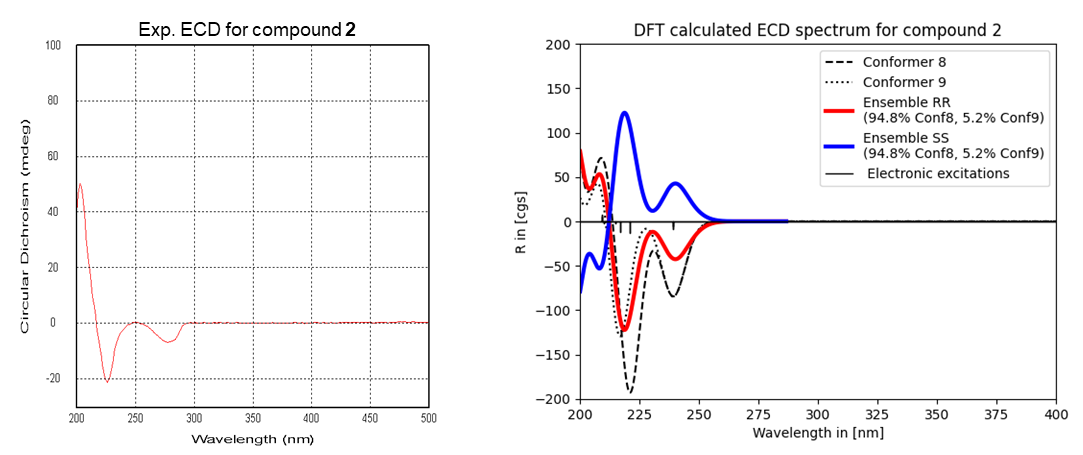
**

**Fig. S13** Experimental and back-calculated ECD spectrum of compound **2**, which reveals the absolute configuration of compound **2** to be 2*R*, 2'*R*. For the TD-DFT calculation, the DFT level ωB97-X/def2-TZVPPD (34) in combination with implicit SMD solvation in methanol was used and 50 roots were selected for the calculation of the electronic excitations.

**1.9 Reference**

1. Meng L-H, Wang C-Y, Mándi A, Li X-M, Hu X-Y, Kassack MU, et al. Three Diketopiperazine Alkaloids with Spirocyclic Skeletons and One Bisthiodiketopiperazine Derivative from the Mangrove-Derived Endophytic Fungus Penicillium brocae MA-231. Org Lett. 2016;18(20):5304-7.

2. Niu S, Liu D, Shao Z, Proksch P, Lin W. Eutypellazines A–M, thiodiketopiperazine-type alkaloids from deep sea derived fungus Eutypella sp. MCCC 3A00281. RSC Advances. 2017;7(53):33580-90.

3. Li X-L, Chi L-P, Navarro-Vázquez A, Hwang S, Schmieder P, Li X-M, et al. Stereochemical Elucidation of Natural Products from Residual Chemical Shift Anisotropies in a Liquid Crystalline Phase. Journal of the American Chemical Society. 2020;142(5):2301-9.

4. Lei X, Qiu F, Sun H, Bai L, Wang W-X, Xiang W, et al. A Self-Assembled Oligopeptide as a Versatile NMR Alignment Medium for the Measurement of Residual Dipolar Couplings in Methanol. Angew Chem Int Ed. 2017;56(42):12857-61.

5. Enthart A, Freudenberger JC, Furrer J, Kessler H, Luy B. The CLIP/CLAP-HSQC: Pure absorptive spectra for the measurement of one-bond couplings. J Magn Reson. 2008;192(2):314-22.

6. Schrödinger L, , . MacroModel. New York, NY.

7. Frisch MJ, Trucks GW, Schlegel HB, Scuseria GE, Robb MA, Cheeseman JR, et al. Gaussian 16 Rev. C.01. Wallingford, CT2016.

8. Tomasi J, Mennucci B, Cammi R. Quantum mechanical continuum solvation models. Chem Rev. 2005;105(8):2999-3093.

9. Denis PA. Basis Set Requirements for Sulfur Compounds in Density Functional Theory:  a Comparison between Correlation-Consistent, Polarized-Consistent, and Pople-Type Basis Sets. J Chem Theory Comput. 2005;1(5):900-7.

10. Wolinski K, Hinton JF, Pulay P. Efficient implementation of the gauge-independent atomic orbital method for NMR chemical shift calculations. Journal of the American Chemical Society. 1990;112(23):8251-60.

11. Navarro-Vázquez A. MSpin-RDC. A program for the use of residual dipolar couplings for structure elucidation of small molecules. Magn Reson Chem. 2012;50 Suppl 1:S73-9.

12. Navarro-Vázquez A. When not to rely on Boltzmann populations. Automated CASE-3D structure elucidation of hyacinthacines through chemical shift differences. Magn Reson Chem. 2020;58(2):139-44.

13. Ehlert S, Grimme S, Hansen A. Conformational Energy Benchmark for Longer n-Alkane Chains. J Phys Chem A. 2022;126(22):3521-35.

14. Johnson ER, Becke AD. A post-Hartree–Fock model of intermolecular interactions. The Journal of Chemical Physics. 2005;123(2).

15. Weigend F, Ahlrichs R. Balanced basis sets of split valence, triple zeta valence and quadruple zeta valence quality for H to Rn: Design and assessment of accuracy. Physical Chemistry Chemical Physics. 2005;7(18):3297-305.

16. Johnson ER, Becke AD. A post-Hartree-Fock model of intermolecular interactions: Inclusion of higher-order corrections. The Journal of Chemical Physics. 2006;124(17).

17. Grimme S, Antony J, Ehrlich S, Krieg H. A consistent and accurate ab initio parametrization of density functional dispersion correction (DFT-D) for the 94 elements H-Pu. The Journal of Chemical Physics. 2010;132(15).

18. Grimme S. Exploration of Chemical Compound, Conformer, and Reaction Space with Meta-Dynamics Simulations Based on Tight-Binding Quantum Chemical Calculations. J Chem Theory Comput. 2019;15(5):2847-62.

19. Grimme S, Bohle F, Hansen A, Pracht P, Spicher S, Stahn M. Efficient Quantum Chemical Calculation of Structure Ensembles and Free Energies for Nonrigid Molecules. J Phys Chem A. 2021;125(19):4039-54.

20. Bannwarth C, Ehlert S, Grimme S. GFN2-xTB-An Accurate and Broadly Parametrized Self-Consistent Tight-Binding Quantum Chemical Method with Multipole Electrostatics and Density-Dependent Dispersion Contributions. J Chem Theory Comput. 2019;15(3):1652-71.

21. Ehlert S, Stahn M, Spicher S, Grimme S. Robust and Efficient Implicit Solvation Model for Fast Semiempirical Methods. J Chem Theory Comput. 2021;17(7):4250-61.

22. Marenich AV, Cramer CJ, Truhlar DG. Universal Solvation Model Based on Solute Electron Density and on a Continuum Model of the Solvent Defined by the Bulk Dielectric Constant and Atomic Surface Tensions. J Phys Chem B. 2009;113(18):6378-96.

23. Spicher S, Grimme S. Single-Point Hessian Calculations for Improved Vibrational Frequencies and Rigid-Rotor-Harmonic-Oscillator Thermodynamics. J Chem Theory Comput. 2021;17(3):1701-14.

24. Troche-Pesqueira E, Anklin C, Gil RR, Navarro-Vázquez A. Computer-Assisted 3D Structure Elucidation of Natural Products using Residual Dipolar Couplings. Angew Chem Int Ed. 2017;56(13):3660-4.

25. Navarro-Vázquez A, Gil RR, Blinov K. Computer-Assisted 3D Structure Elucidation (CASE-3D) of Natural Products Combining Isotropic and Anisotropic NMR Parameters. J Nat Prod. 2018;81(1):203-10.

26. Losonczi JA, Andrec M, Fischer MWF, Prestegard JH. Order Matrix Analysis of Residual Dipolar Couplings Using Singular Value Decomposition. J Magn Reson. 1999;138(2):334-42.

27. Neese F, Wennmohs F, Becker U, Riplinger C. The ORCA quantum chemistry program package. J Chem Phys. 2020;152(22):224108.

28. Neese F, Wennmohs F, Becker U, Riplinger C. The ORCA quantum chemistry program package. The Journal of Chemical Physics. 2020;152(22).

29. Lu T, Chen F. Multiwfn: A multifunctional wavefunction analyzer. J Comput Chem. 2012;33(5):580-92.

30. Lu T, Chen Q. Independent gradient model based on Hirshfeld partition: A new method for visual study of interactions in chemical systems. J Comput Chem. 2022;43(8):539-55.

31. Humphrey W, Dalke A, Schulten K. VMD: Visual molecular dynamics. J Mol Graphics. 1996;14(1):33-8.

32. Autschbach J, Nitsch-Velasquez L, Rudolph M. Time-dependent density functional response theory for electronic chiroptical properties of chiral molecules. Top Curr Chem. 2011;298:1-98.

33. Pescitelli G, Bruhn T. Good Computational Practice in the Assignment of Absolute Configurations by TDDFT Calculations of ECD Spectra. Chirality. 2016;28(6):466-74.

34. Chai JD, Head-Gordon M. Systematic optimization of long-range corrected hybrid density functionals. J Chem Phys. 2008;128(8):084106.

**2** **Additional figures** **of spectroscopic spectra of compounds 1 and 2**

**Fig. S12** HR-ESI-MS spectrum of compound **1**.

**Fig. S13** ^1^H NMR spectrum of compound **1** (500 MHz, DMSO-*d*_6_).

**Fig. S14** ^13^C NMR and DEPT spectrum of compound **1** (125 MHz, DMSO-*d*_6_).

**Fig. S15** COSY spectrum of compound **1** (500 MHz, DMSO-*d*_6_).

**Fig. S16** HMBC spectrum of compound **1** (500 MHz, DMSO-*d*_6_).

**Fig. S17** NOESY spectrum of compound **1** (500 MHz, DMSO-*d*_6_).

**Fig. S18** HR-ESI-MS spectrum of compound **2**.

**Fig. S19** ^1^H NMR spectrum of compound **2** (500 MHz, DMSO-*d*_6_).

**Fig. S20** ^13^C NMR and DEPT spectrum of compound **2** (125 MHz, DMSO-*d*_6_).

**Fig. S21** COSY spectrum of compound **2** (500 MHz, DMSO-*d*_6_).

**Fig. S22** HMBC spectrum of compound **2** (500 MHz, DMSO-*d*_6_).

**Fig.** **S23** NOESY spectrum of compound **2** (500 MHz, DMSO-*d*_6_).

**
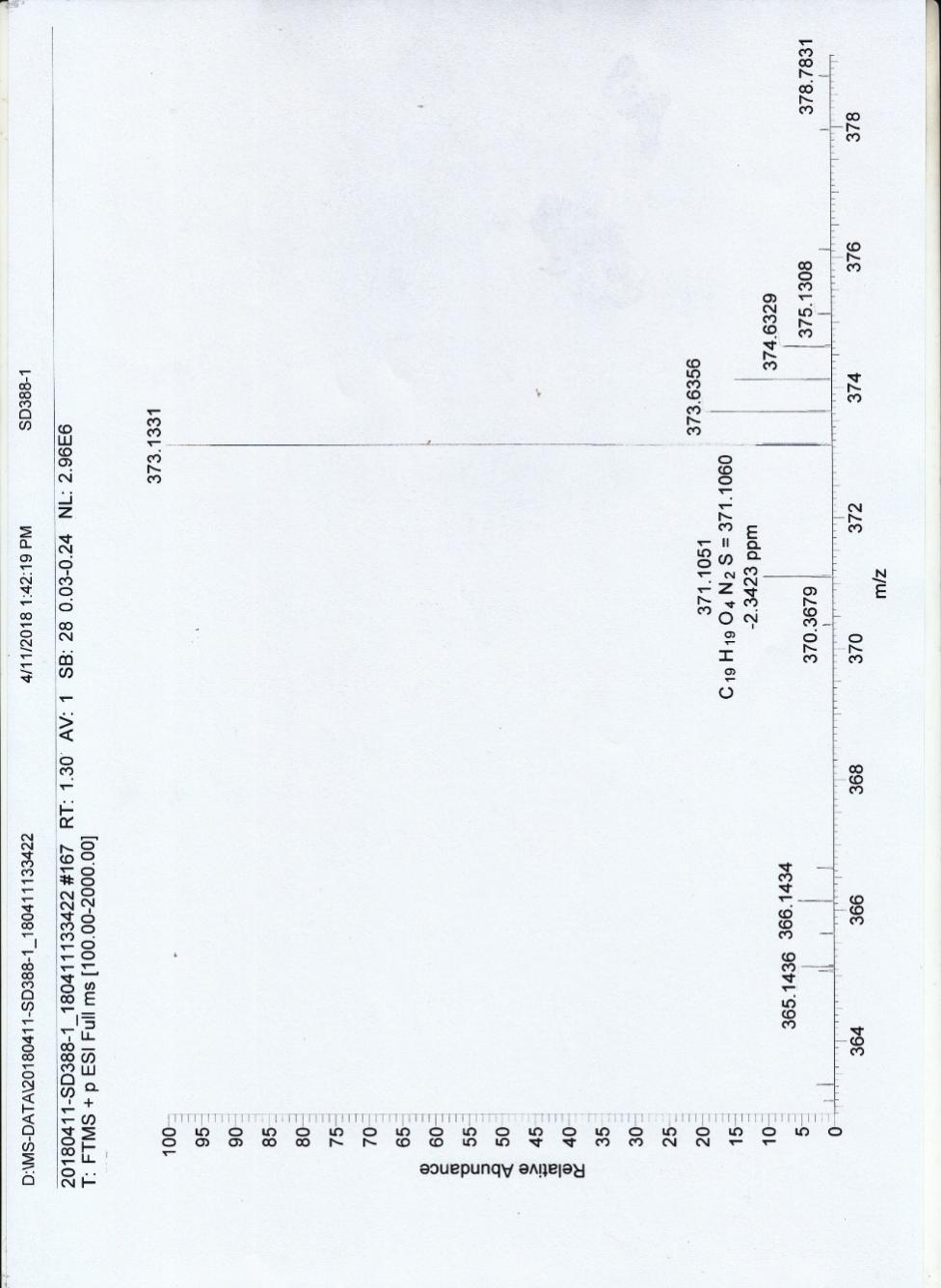
**

**Fig. S12** HR-ESI-MS spectrum of compound **1**.

**Fig. S13** ^1^H NMR spectrum of compound **1** (500 MHz, DMSO-*d*_6_).

**Fig. S14** ^13^C NMR and DEPT spectrum of compound **1** (125 MHz, DMSO-*d*_6_).

**Fig. S15** COSY spectrum of compound **1** (500 MHz, DMSO-*d*_6_).

**Fig. S16** HMBC spectrum of compound **1** (500 MHz, DMSO-*d*_6_).

**Fig. S17** NOESY spectrum of compound **1** (500 MHz, DMSO-*d*_6_).

**
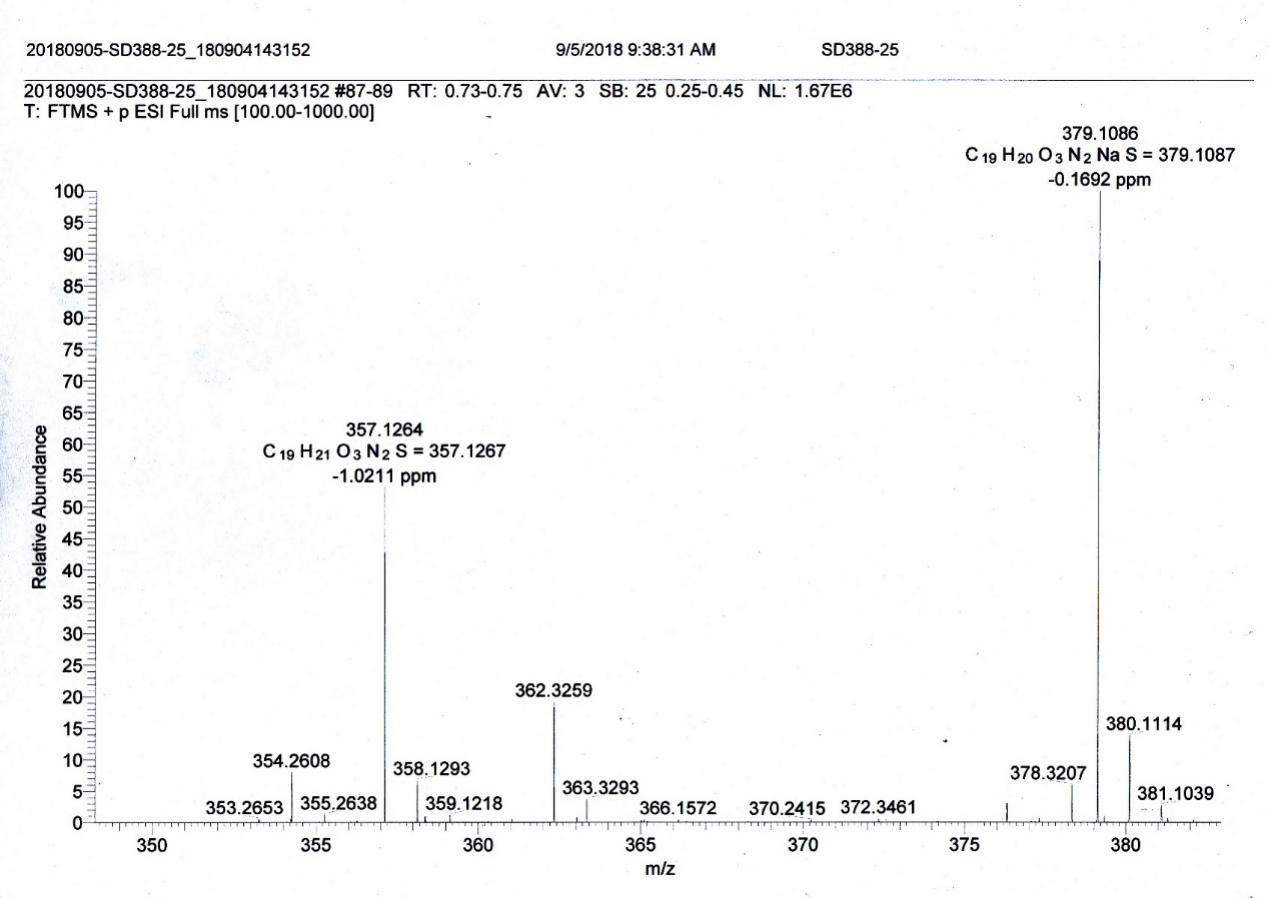
Fig. S18** HR-ESI-MS spectrum of compound **2**.

**Fig. S19** ^1^H NMR spectrum of compound **2** (500 MHz, DMSO-*d*_6_).

**Fig. S20** ^13^C NMR and DEPT spectrum of compound **2** (125 MHz, DMSO-*d*_6_).

**Fig. S21** COSY spectrum of compound **2** (500 MHz, DMSO-*d*_6_).

**Fig. S22** HMBC spectrum of compound **2** (500 MHz, DMSO-*d*_6_).

**Fig. S23** NOESY spectrum of compound **2** (500 MHz, DMSO-*d*_6_).
